# Association of Net Ultrafiltration Rate With Mortality Among Critically Ill Adults With Acute Kidney Injury Receiving Continuous Venovenous Hemodiafiltration: A Secondary Analysis of the Randomized Evaluation of Normal vs Augmented Level (RENAL) of Renal Replacement Therapy Trial

**DOI:** 10.1001/jamanetworkopen.2019.5418

**Published:** 2019-06-07

**Authors:** Raghavan Murugan, Samantha J. Kerti, Chung-Chou H. Chang, Martin Gallagher, Gilles Clermont, Paul M. Palevsky, John A. Kellum, Rinaldo Bellomo

**Affiliations:** 1 The Clinical Research, Investigation, and Systems Modeling of Acute Illness (CRISMA) Center, Department of Critical Care Medicine, University of Pittsburgh School of Medicine, Pittsburgh, Pennsylvania; 2 The Center for Critical Care Nephrology, Department of Critical Care Medicine, University of Pittsburgh School of Medicine, Pittsburgh, Pennsylvania; 3 Department of Biostatistics, Graduate School of Public Health, University of Pittsburgh, Pittsburgh, Pennsylvania; 4 Department of Medicine, University of Pittsburgh School of Medicine, Pittsburgh, Pennsylvania; 5 The George Institute for Global Health and University of Sydney, Sydney, New South Wales, Australia; 6 Renal Section, Veterans Affairs Pittsburgh Healthcare System, Pittsburgh, Pennsylvania; 7 Department of Intensive Care Medicine, Austin Hospital, The University of Melbourne, Melbourne, Victoria, Australia

## Abstract

**Importance:**

Net ultrafiltration (NUF) is frequently used to treat fluid overload among critically ill patients, but whether the rate of NUF affects outcomes is unclear.

**Objective:**

To examine the association of NUF with survival among critically ill patients with acute kidney injury being treated with continuous venovenous hemodiafiltration.

**Design, Setting, and Participants:**

The Randomized Evaluation of Normal vs Augmented Level (RENAL) of Renal Replacement Therapy trial was conducted between December 30, 2005, and November 28, 2008, at 35 intensive care units in Australia and New Zealand among critically ill adults with acute kidney injury who were being treated with continuous venovenous hemodiafiltration. This secondary analysis began in May 2018 and concluded in January 2019.

**Exposures:**

Net ultrafiltration rate, defined as the volume of fluid removed per hour adjusted for patient body weight.

**Main Outcomes and Measures:**

Risk-adjusted 90-day survival.

**Results:**

Of 1434 patients, the median (interquartile range) age was 67.3 (56.9-76.3) years; 924 participants (64.4%) were male; median (interquartile range) Acute Physiology and Chronic Health Evaluation III score was 100 (84-118); and 634 patients (44.2%) died. Using tertiles, 3 groups were defined: high, NUF rate greater than 1.75 mL/kg/h; middle, NUF rate from 1.01 to 1.75 mL/kg/h; and low, NUF rate less than 1.01 mL/kg/h. The high-tertile group compared with the low-tertile group was not associated with death from day 0 to 6. However, death occurred in 51 patients (14.7%) in the high-tertile group vs 30 patients (8.6%) in the low-tertile group from day 7 to 12 (adjusted hazard ratio [aHR], 1.51; 95% CI, 1.13-2.02); 45 patients (15.3%) in the high-tertile group vs 25 patients (7.9%) in the low-tertile group from day 13 to 26 (aHR, 1.52; 95% CI, 1.11-2.07); and 48 patients (19.2%) in the high-tertile group vs 29 patients (9.9%) in the low-tertile group from day 27 to 90 (aHR, 1.66; 95% CI, 1.16-2.39). Every 0.5-mL/kg/h increase in NUF rate was associated with increased mortality (3-6 days: aHR, 1.05; 95% CI, 1.00-1.11; 7-12 days: aHR, 1.08; 95% CI, 1.02-1.15; 13-26 days: aHR, 1.11; 95% CI, 1.04-1.18; 27-90 days: aHR, 1.13; 95% CI, 1.05-1.22). Using longitudinal analyses, increase in NUF rate was associated with lower survival (β = .056; *P* < .001). Hypophosphatemia was more frequent among patients in the high-tertile group compared with patients in the middle-tertile group and patients in the low-tertile group (high: 308 of 477 patients at risk [64.6%]; middle: 293 of 472 patients at risk [62.1%]; low: 247 of 466 patients at risk [53.0%]; *P* < .001). Cardiac arrhythmias requiring treatment occurred among all groups: high, 176 patients (36.8%); middle: 175 patients (36.5%); and low: 147 patients (30.8%) (*P* = .08).

**Conclusions and Relevance:**

Among critically ill patients, NUF rates greater than 1.75 mL/kg/h compared with NUF rates less than 1.01 mL/kg/h were associated with lower survival. Residual confounding may be present from unmeasured risk factors, and randomized clinical trials are required to confirm these findings.

**Trial Registration:**

ClinicalTrials.gov identifier: NCT00221013

## Key Points

**Question:**

Is the net ultrafiltration (ie, fluid removal) rate associated with survival among critically ill patients with acute kidney injury?

**Findings:**

In this secondary analysis of a randomized clinical trial involving 1434 critically ill patients treated with continuous venovenous hemodiafiltration, a net ultrafiltration rate greater than 1.75 mL/kg/h compared with a net ultrafiltration rate less than 1.01 mL/kg/h was significantly associated with lower 90-day risk-adjusted survival.

**Meaning:**

Among critically ill patients with acute kidney injury being treated with continuous venovenous hemodiafiltration, net ultrafiltration rates greater than 1.75 mL/kg/h were associated with increased mortality.

## Teaser

This secondary analysis of the Randomized Evaluation of Normal vs Augmented Level (RENAL) of Renal Replacement Therapy trial examines the association of net ultrafiltration with survival among critically ill patients with acute kidney injury being treated with continuous venovenous hemodiafiltration.

## Introduction

Fluid overload is a frequent complication present in more than two-thirds of critically ill patients with acute kidney injury and is independently associated with mortality.^1,2^ When fluid overload is resistant to treatment with diuretics, international practice guidelines recommend net ultrafiltration (NUF).^3,4^ These recommendations are supported by studies suggesting that NUF could reduce the number of deaths.^5,6^ However, uncertainty exists about the optimal rate of NUF in critically ill patients.

A slower NUF rate is associated with prolonged exposure to tissue edema and organ dysfunction, whereas a faster rate is associated with hemodynamic stress.^7,8^ Both complications could decrease survival. A single-center observational study of critically ill patients receiving continuous venovenous hemodiafiltration (CVVHDF) and hemodialysis^9^ found that an NUF rate less than 20 mL/kg/d was associated with higher mortality compared with an NUF rate greater than 25 mL/kg/d. In contrast, emerging evidence from outpatients with end-stage renal disease receiving hemodialysis suggests that an NUF rate greater than 13 mL/kg/h per session compared with an NUF rate of 10 mL/kg/h or less is associated with mortality.^10,11,12,13^ However, the implications and generalizability of these study findings to patients undergoing continuous NUF are unclear.

Thus, we performed a secondary analysis of the Randomized Evaluation of Normal vs Augmented Level (RENAL) of Renal Replacement Therapy clinical trial^14^ of critically ill patients treated with CVVHDF. In this study, we examine the association of NUF rate with risk-adjusted 90-day survival as well as adverse events during treatment.

## Methods

We followed the Strengthening the Reporting of Observational Studies in Epidemiology (STROBE) reporting guideline.^15^ This retrospective cohort study was approved with a waiver of informed consent by the University of Pittsburgh’s Human Research Protection Office. Written informed consent was obtained from the patient or responsible surrogate by means of either a priori or delayed consent in the RENAL trial.^14^

### Population

The RENAL study was a multicenter randomized clinical trial that compared the efficacy of 2 different intensities of solute control using CVVHDF in critically ill patients with acute kidney injury.^14^ The study was conducted in 35 intensive care units (ICUs) in Australia and New Zealand from December 30, 2005, to November 28, 2008. This secondary analysis was performed from May 31, 2018, to January 31, 2019. In brief, patients were eligible to participate in the study if they were critically ill adults with acute kidney injury, were deemed to require CVVHDF by a clinician, and fulfilled predefined criteria, including oliguria, severe organ edema, hyperkalemia, uremia, and/or severe metabolic acidosis (eAppendix 1 in the Supplement). The NUF rate was left to clinician judgment and was performed by decreasing the flow of the replacement fluid and the dialysate in equal proportion, so that effluent fluid volume exceeded replacement fluid and dialysate volumes.

### Variables

The primary outcome was 90-day survival from study enrollment. The exposure variable was NUF rate, defined as the volume of net ultrafiltrate removed per hour, adjusted for patient body weight in kilograms. The hourly NUF volume was calculated after excluding the dialysate and replacement fluid volumes from the volume of ultrafiltrate (ie, NUF volume = ultrafiltrate volume − [replacement fluid + dialysate volume]). Subsequently, rate for duration of treatment was calculated using the following equation^9^: NUF rate (in milliliters per kilogram per hour) = cumulative NUF volume (in milliliters) / (weight at study enrollment [in kilograms] × treatment duration [in hours]). Daily patient fluid balance was first calculated as the difference between fluid administered (ie, intravenous fluids, blood products, enteral fluids, dialysate, and replacement fluid) and fluid lost (ie, dialysis effluent from CVVHDF, urine output, blood loss, enteral losses, and drain losses). We then excluded NUF volume from the output fluids since it was the exposure variable.^9^ Daily and cumulative fluid balance data were obtained from study enrollment until death, ICU discharge, or 28 days after enrollment. Complications and other adverse events were recorded during treatment.^14^

We measured covariates for a risk-adjustment model to account for confounding by indication^16^ because patients who were older, sicker, and hemodynamically unstable would be expected to receive a lower rate and patients with organ edema and those requiring mechanical ventilation would be expected to receive a higher rate. These confounders included prespecified variables based on clinical experience and prior studies,^5,6,9^ including age category; female sex; premorbid estimated glomerular filtration rate (eGFR) based on most recent serum creatinine level, if known; duration from ICU admission to study enrollment; severity of illness assessed by Acute Physiology and Chronic Health Evaluation III (APACHE-III) score category (range, 0-299, with higher score indicating more severe illness) in the 24 hours prior to study enrollment; severity of organ dysfunction assessed by total Sequential Organ Failure Assessment (SOFA) score (range, 0-4 for each organ, with higher score indicating more severe organ dysfunction); presence of organ edema, sepsis, and use of mechanical ventilation; daily mean cardiovascular SOFA score during treatment; cumulative fluid balance from enrollment to ICU discharge; duration of CVVHDF in days; source of admission, including whether the patient was transferred from an emergency department, hospital ward, operating room after elective or emergency surgery, another hospital, or another ICU; hospital type; and hospital region. Race and ethnicity were not reported in the randomized clinical trial.

For patients with unknown premorbid serum creatinine levels (637 [44.4%]), we used the multivariable imputation by chained equation method to impute creatinine values using age, sex, and weight as predictors (eAppendix 2 in the Supplement).^17,18^ We subsequently used the Modification of Diet in Renal Disease Study equation to determine eGFR using the imputed creatinine levels.^19^ There was no difference in distribution of imputed and unimputed creatinine and corresponding eGFR values (eFigure 1 in the Supplement).

### Statistical Analysis

We examined several models, including linear, spline, median, tertiles, and quartiles, because of the nonlinear association of NUF rate with 90-day mortality (eFigure 2 in the Supplement). We selected tertiles owing to having the lowest Akaike information criterion. Thus, we stratified NUF rates into 3 groups: (1) low, less than 1.01 mL/kg/h; (2) middle, 1.01 to 1.75 mL/kg/h; and (3) high, greater than 1.75 mL/kg/h. Categorical variables are presented as numbers and percentages and compared using χ^2^ tests. Continuous variables are presented as medians and interquartile ranges (IQRs) and compared using the Wilcoxon rank sum test.

Multivariable modeling of the association of NUF rate with survival was performed using Gray piecewise-constant time-varying coefficients regression (eAppendix 3 and eFigure 3 in the Supplement).^20,21,22,23^ We estimated risk-adjusted hazard ratios (aHRs) and their 95% CIs at 5 time intervals and 4 nodes (0-2 days, 3-6 days, 7-12 days, 13-26 days, and 27-90 days). The number of time intervals were selected based on prior work,^9^ and the duration of each time interval was selected by the model to ensure approximately equal distribution of deaths within each time interval (eTable 1 in the Supplement).^21^

In these models, we used an NUF rate less than 1.01 mL/kg/h as the reference and adjusted for covariates with fixed effects for region and hospital type to account for nonindependence of NUF across hospitals. Models were fitted using 1341 patients (93.5%) after excluding patients with missing covariate data on source of ICU admission (91 [6.3%]), number of CVVHDF treatment days (1 [0.1%]), and mortality (1 [0.1%]). To predict the risk of death across a range of NUF rates, we restricted the cohort to NUF rate of 5 mL/kg/h or less (1428 patients [99.6%]). We also fitted similar models for patients with only available premorbid serum creatinine levels (797 [55.6%]). We performed longitudinal analyses using joint models to account for correlation between daily NUF rate and cardiovascular SOFA score over time and its association with survival (eAppendix 4 in the Supplement).^24,25^

We assessed the robustness of findings in multiple sensitivity analyses. First, using propensity scores, we matched patients with NUF rates greater than 1.75 mL/kg/h on a 1:1 basis with patients with NUF rates of 1.75 mL/kg/h or less (eAppendix 5 and eFigure 4 in the Supplement). Second, we examined alternative thresholds by lowering and increasing the values by 0.05 mL/kg/h (ie, <0.96 mL/kg/h, 0.96-1.70 mL/kg/h, >1.70 mL/kg/h and <1.06 mL/kg/h, 1.06-1.80 mL/kg/h, >1.80 mL/kg/h). Third, we restricted rate to the first 72 hours of CVVHDF. Fourth, we classified patients using maximum NUF rate.

Fifth, we excluded 92 patients with NUF rates less than 0.01 mL/kg/h. Sixth, we included 31 patients with missing treatment duration data by assigning 2 NUF rates (0 mL/kg/h and 1.43 mL/kg/h [the mean NUF rate]) before fitting the model. Seventh, we varied the time interval by moving the nodes in the Gray model 1 day higher (0-3 days, 4-7 days, 8-13 days, 14-27 days, and 28-90 days) and 1 day lower (0-1 day, 2-5 days, 6-11 days, 12-25 days, and 26-90 days). Eighth, we adjusted for individual baseline liver, coagulation, and respiratory SOFA scores instead of total SOFA scores. Ninth, we adjusted for use of red blood cells, fresh frozen plasma, platelets, cryoprecipitate, 20% albumin, and cumulative protein supplementation. Tenth, we adjusted for cumulative fluid balance that included NUF volume in output fluid calculation. Eleventh, we excluded cumulative fluid balance from the model.

Twelfth, we stratified based on CVVHDF for less than 3 days and 3 or more days as well as less than 5 days and 5 or more days and among patients with negative daily fluid balance during ICU stay. Thirteenth, we fitted logistic regression with rate as a categorical variable after excluding collinearity using variance inflation factor (eAppendix 6 and eFigure 5 in the Supplement). To predict the risk of death across a range of rates, we restricted the analysis to NUF rates of 5 mL/kg/h or greater and used predictive margins adjusted for covariates to predict death for every 0.5-mL/kg/h increase in rate (eFigure 6 in the Supplement). Using subgroup analyses, we assessed for effect modification with a test for interaction in the Gray model between NUF rate and patient characteristics in prespecified subpopulations, including patients with and without organ edema; sepsis; premorbid eGFR less than 60 mL/min/1.73m^2 ^and 60 mL/min/1.73m^2^ or greater; cardiovascular SOFA score less than 3 and 3 or higher; and high and low intensity CVVHDF.

Statistical analyses were performed using SAS 9.4 (SAS Institute) and Stata version 15.1 for Windows (StataCorp). Gray and joint models were performed using R version 2.14.0 (The R Foundation) and multiple imputation using the MICE command in R version 3.4.2. All hypothesis tests were 2-tailed with a statistical significance level of *P* < .05.

## Results

### Patient Population and Characteristics

Of 1508 patients enrolled in the RENAL trial,^14^ consent was withdrawn by 43 patients. Of the remaining 1465 patients, we excluded 31 patients for whom the treatment hours for CVVHDF were missing (eTable 2 in the Supplement). Of 1434 eligible patients, 477 patients (33.3%) received NUF less than 1.01 mL/kg/h, 479 patients (33.4%) received NUFfrom 1.01 to 1.75 mL/kg/h, and 478 patients (33.3%) received NUF greater than 1.75 mL/kg/h. Median (IQR) age was 67.3 (56.9-76.3) years; median (IQR) body weight was 80.0 (70.0-90.0) kg; and 924 patients (64.4%) were men (Table 1). Median (IQR) premorbid eGFR was 53.0 (32.6-73.9) mL/min/1.73 m^2^; median (IQR) APACHE-III score was 100 (84-118); and 634 patients (44.2%) died. Median (IQR) NUF rates within the 3 tertiles were as follows: 0.52 (0.06-0.79) mL/kg/h in the lowest tertile; 1.38 (1.19-1.55) mL/kg/h in the middle tertile; and 2.21 (1.96-2.68) mL/kg/h in the highest tertile (mean [SD] NUF rate, 1.43 [0.97] mL/kg/h).

**Table 1.** Baseline Patient Characteristics by NUF Rate

Characteristic	No. (%)				*P* Value
	All Patients	NUF Rate <1.01 mL/kg/h	NUF Rate 1.01-1.75 mL/kg/h	NUF Rate >1.75 mL/kg/h	
Total, No.	1434	477	479	478	NA
Age, median (IQR), y	67.3 (56.9-76.3)	69.3 (61.0-77.4)	68.1 (57.2-76.1)	63.8 (51.4-74.2)	<.001
Age category, y					
<53.2	287 (20.0)	65 (13.6)	96 (20.0)	126 (26.4)	<.001
53.2 to <63.6	287 (20.0)	93 (19.5)	83 (17.3)	111 (23.3)	
63.6 to <71.1	286 (19.9)	98 (20.5)	97 (20.2)	91 (19.0)	
71.1 to <77.7	287 (20.0)	107 (22.4)	111 (23.2)	69 (14.4)	
≥77.7	287 (20.0)	114 (23.9)	92 (19.2)	81 (17.0)	
Men	924 (64.4)	311 (65.2)	330 (68.9)	283 (59.2)	.007
Weight, median (IQR), kg^a^	80.0 (70.0-90.0)	81.0 (73.0-90.0)	80.0 (74.0-90.0)	75.0 (68.0-85.0)	<.001
Preadmission eGFR, median (IQR), mL/min/1.73m^2^^b^	53.0 (32.6-73.9)	48.1 (31.0-68.4)	51.0 (34.0-72.8)	59.0 (33.3-80.2)	.009
No. of patients with known eGFR, mL/min/1.73 m^2^^b^	467	178	150	139	NA
46 to <60	143 (30.6)	52 (29.2)	48 (32.0)	43 (30.9)	.63
30 to <46	156 (33.4)	64 (36.0)	52 (34.7)	40 (28.8)	
<30	168 (36.0)	62 (34.8)	50 (33.3)	56 (40.3)	
Time in ICU before randomization, median (IQR), h	20.0 (6.0-51.0)	13.0 (3.0-41.0)	21.0 (6.0-53.0)	26.0 (8.0-63.0)	<.001
Mechanical ventilation^a^	1057 (73.7)	330 (69.2)	356 (74.3)	371 (77.6)	.01
Sepsis^a^	709 (49.4)	221 (46.3)	235 (49.1)	253 (53.0)	.12
APACHE-III score, median (IQR)^c^^,^^d^	100 (84-118)	101 (84-118)	100 (83-118)	101 (84-117)	.94
APACHE-III category^c^^,^^d^					
<82	273 (19.0)	87 (18.2)	95 (19.8)	91 (19.0)	.99
82 to <95	299 (20.9)	103 (21.6)	99 (20.7)	97 (20.3)	
95 to <107	277 (19.3)	92 (19.3)	88 (18.4)	97 (20.3)	
107 to <122	289 (20.1)	97 (20.3)	96 (20.0)	96 (20.1)	
≥122	296 (20.6)	98 (20.6)	101 (21.1)	97 (20.3)	
SOFA score, median (IQR)^c^^,^^e^					
Total	8 (6-9)	7 (5-9)	8 (6-9)	8 (6-10)	.001
Individual					
Cardiovascular	4 (1-4)	4 (1-4)	4 (2-4)	4 (1-4)	.90
Respiratory	3 (2-3)	3 (2-3)	3 (2-3)	3 (3-3)	.007
Coagulation	0 (0-2)	0 (0-2)	0 (0-2)	1.0 (0-2)	.002
Liver	0 (0-2)	0 (0-2)	0 (0-2)	1.0 (0-2)	.001
Source of admission					
Emergency department	341 (25.4)	139 (31.1)	117 (26.1)	85 (17.8)	<.001
Hospital ward	378 (28.1)	114 (25.5)	111 (24.7)	153 (34.2)	
Another ICU	109 (8.1)	29 (6.5)	40 (8.9)	40 (8.9)	
Another hospital	149 (11.1)	56 (12.5)	47 (10.5)	46 (10.3)	
OR after emergency surgery	204 (15.2)	68 (15.2)	71 (15.8)	65 (14.5)	
OR after elective surgery	162 (12.1)	41 (9.2)	63 (13.2)	58 (12.1)	
Criteria for randomization^c^^,^^f^					
Oliguria	855 (59.6)	277 (58.1)	292 (61.0)	286 (59.8)	.65
Hyperkalemia	111 (7.7)	54 (11.3)	33 (6.9)	24 (5.0)	<.001
Severe acidemia	506 (35.3)	211 (44.2)	141 (29.4)	154 (32.2)	<.001
BUN >70 mg/dL	595 (41.5)	205 (43.0)	197 (41.1)	193 (40.4)	.70
Creatinine >3.4 mg/dL	679 (47.4)	248 (52.0)	238 (49.7)	193 (40.4)	<.001
Severe organ edema due to kidney disease	634 (44.2)	172 (36.1)	207 (43.2)	255 (53.3)	<.001
Year of enrollment					
2005	1 (0.1)	0	1 (0.2)	0	.82
2006	305 (21.3)	99 (20.7)	108 (22.5)	98 (20.5)	
2007	629 (43.9)	209 (43.8)	205 (42.8)	215 (45.0)	
2008	499 (34.8)	169 (35.4)	165 (34.4)	165 (34.5)	
Type of hospital					
University	1023 (71.3)	313 (65.6)	338 (70.6)	372 (77.8)	<.001
Urban	338 (23.6)	122 (25.6)	121 (25.3)	95 (19.9)	
Rural	23 (1.6)	16 (3.3)	7 (1.5)	0	
Private	50 (3.5)	26 (5.4)	13 (2.7)	11 (2.3)	
Country					
New Zealand	104 (7.3)	55 (11.5)	28 (5.8)	21 (4.4)	<.001
Australia	1330 (92.8)	422 (88.5)	451 (94.1)	457 (95.6)	
Region					
New Zealand	104 (7.3)	55 (11.5)	28 (5.8)	21 (0.4)	<.001
New South Wales, Australia	523 (36.5)	143 (30.0)	183 (38.2)	197 (41.2)	
Queensland, Australia	85 (5.9)	27 (5.6)	19 (4.0)	39 (8.2)	
Victoria, Australia	578 (40.3)	195 (40.9)	205 (42.8)	178 (37.3)	
Western Australia, Australia	65 (4.5)	21 (4.4)	22 (4.6)	22 (4.6)	
Tasmania, Australia	18 (1.3)	7 (1.5)	4 (0.9)	7 (1.5)	
Australian Capital Territory, Australia	6 (0.4)	3 (0.6)	1 (0.2)	2 (0.4)	
South Australia, Australia	55 (3.8)	26 (5.4)	17 (3.5)	12 (2.5)	

Abbreviations: APACHE-III, Acute Physiology and Chronic Health Evaluation III; BUN, blood urea nitrogen; eGFR, estimated glomerular filtration rate; ICU, intensive care unit; IQR, interquartile range; NA, not applicable; NUF, net ultrafiltration; OR, operating room; SOFA, Sequential Organ Failure Assessment.

SI conversion factors: To convert creatinine level to micromoles per liter, multiply by 88.4; BUN to millimoles per liter, multiply by 0.357.

^a^ Measured at study enrollment.

^b^ Data are for 797 patients for whom the eGFR before study enrollment was known.

^c^ Measured 24 hours prior to study enrollment.

^d^ Scores on APACHE-III range from 0 to 299, with higher scores indicating more severe illness.

^e^ Scores on SOFA range from 0 to 4, with a higher score indicating more severe organ dysfunction.

^f^ A given patient may have met multiple criteria.

Net ultrafiltration rates greater than 1.75 mL/kg/h were associated with young age, female sex, lower body weight, higher eGFR, mechanical ventilation, and longer ICU stay than patients receiving NUF rates from 1.01 to 1.75 mL/kg/h or less than 1.01 mL/kg/h. Patients with NUF rates greater than 1.75 mL/kg/h had more severe organ dysfunction, as evidenced by higher median (IQR) total SOFA score (high: 8 [6-10]; middle: 8 [6-9]; low: 7 [5-9]; *P* = .001) and organ edema (high: 255 patients [53.3%]; middle: 207 patients [43.2%]; low: 172 patients [36.1%]; *P* < .001). There were also variations in source of admission to the ICU, type of hospital, country, and region (Table 1).

Following initiation of CVVHDF, patients with NUF rates greater than 1.75 mL/kg/h were associated with having a similar median (IQR) cardiovascular SOFA score (high: 3 [1 to 4]; middle: 3 [1 to 4]; low: 3 [1 to 4]; *P* = .05; Table 2) and longer median (IQR) duration of treatment with CVVHDF (high: 7 [4 to 17] days; middle: 6 [3 to 11] days; low: 3 [2 to 6] days; *P* < .001). Patients receiving NUFgreater than 1.75 mL/kg/h had higher negative median (IQR) daily fluid balances (high: −658.0 [−1445.0 to 55.0] mL/d ; middle: −55.5 [−678.5 to 490.0] mL/d; low: 641.0 [−91.0 to 1793.0] mL/d; *P* < .001) and median (IQR) cumulative fluid balances (high: −3.6 [−7.7 to 0.4] L; middle: −0.4 [−3.5 to 2.4] L; low: 2.3 [−0.2 to 5.5] L; *P* < .001). Median (IQR) hourly NUF rates and cumulative NUF rates were also higher for patients receiving NUFgreater than 1.75 mL/kg/h for the duration of CVVHDF (hourly NUF rate: high: 167.4 [141.5 to 203.0] mL/h; middle: 106.0 [89.5 to 131.0] mL/h; low: 31.8 [0.7 to 62.5] mL/h; *P* < .001; cumulative NUF rate: high: 16.5 [8.5 to 28.4] L; middle: 8.5 [4.5 to 16.4] L; low: 1.7 [0.1 to 4.0] L; *P* < .001).

**Table 2.** Processes of Care During NUF and Outcomes

Characteristic	No. (%)				*P* Value
	All Patients	NUF Rate <1.01 mL/kg/h	NUF Rate 1.01-1.75 mL/kg/h	NUF Rate >1.75 mL/kg/h	
Total, No.	1434	477	479	478	NA
Intensity of RRT					
High	708 (49.4)	244 (51.1)	216 (45.1)	248 (51.8)	.07
Low	726 (50.6)	233 (48.8)	263 (54.9)	230 (48.1)	
Total SOFA score, median (IQR)	8.0 (6.0 to 11.0)	8.0 (6.0 to 11.0)	8.0 (6.0 to 10.0)	8.0 (6.0 to 11.0)	.19
Individual daily mean SOFA score, median (IQR)					
Cardiovascular	3.0 (1.0 to 4.0)	3.0 (1.0 to 4.0)	3.0 (1.0 to 4.0)	3.0 (1.0 to 4.0)	.05
Respiratory	3.0 (3.0 to 3.0)	3.0 (2.0 to 3.0)	3.0 (3.0 to 3.0)	3.0 (3.0 to 3.0)	.18
Coagulation	1.0 (0 to 2.0)	1.0 (0 to 2.0)	1.0 (0 to 2.0)	1.3 (0 to 2.5)	.24
Liver	1.0 (0 to 2.0)	1.0 (0 to 2.0)	1.0 (0 to 2.0)	1.0 (0 to 2.0)	.12
Duration of study treatment, median (IQR), d	3 (2 to 7)	2 (1 to 4)	4 (2 to 8)	5 (2 to 10)	<.001
Effluent flow rate, median (IQR), mL/kg/h	25.0 (25.0 to 40.0)	40.0 (25.0 to 40.0)	25.0 (25.0 to 40.0)	40 (25.0 to 40.0)	.07
Fluid balance, median (IQR)					
Daily, mL/d	−39.5 (−812.0 to 721.0)	641.0 (−91.0 to 1793.0)	−55.5 (−678.5 to 490.0)	−658.0 (−1445.0 to 55.0)	<.001
Cumulative, L	−0.1 (−4.1 to 3.1)	2.3 (−0.2 to 5.5)	−0.4 (−3.5 to 2.4)	−3.6 (−7.7 to 0.4)	<.001
NUF rate, median (IQR)					
Hourly, mL/h	104.7 (56.2 to 150.3)	31.8 (0.7 to 62.5)	106.0 (89.5 to 131.0)	167.4 (141.5 to 203.0)	<.001
Cumulative, L	7.0 (2.4 to 17.0)	1.7 (0.1 to 4.0)	8.5 (4.5 to 16.4)	16.5 (8.5 to 28.4)	<.001
Fluid balance excluding NUF, median (IQR)^a^					
Daily, mL/d	1740.3 (971.0 to 2563.0)	1370.0 (561.0 to 2259.0)	1683.0 (989.0 to 2440.5)	2058.5 (1385.0 to 2882.0)	<.001
Cumulative, L	7.4 (3.1 to 15.3)	4.6 (1.3 to 8.7)	8.5 (3.6 to 15.9)	11.1 (5.1 to 22.4)	<.001
Duration of mechanical ventilation, median (IQR), d	5 (2 to 10)	3 (1 to 7)	6 (2 to 12)	7 (3 to 12)	<.001
Length of stay, median (IQR), d					
ICU	7 (3 to 14)	5 (2 to 10)	8 (4 to 15)	9 (5 to 16)	<.001
Hospital	8 (5 to 15)	6 (3 to 11)	9 (5 to 16)	10 (6 to 16)	<.001
No. of RRTs, median (IQR), d	5 (3 to 11)	3 (2 to 6)	6 (3 to 11)	7 (4 to 17)	<.001
RRT dependence among survivors, No./total No. (%)					
Day 28	120/900 (13.3)	19/290 (6.6)	37/317 (11.7)	64/292 (21.9)	<.001
Day 90	44/800 (5.5)	10/263 (3.8)	17/291 (5.8)	17/246 (6.9)	.28
Death					
Day 28	534 (37.2)	187 (39.2)	162 (33.6)	186 (38.9)	.13
Day 90	634 (44.2)	214 (44.9)	188 (39.2)	232 (48.6)	.01

Abbreviations: ICU, intensive care unit; IQR, interquartile range; NA, not applicable; NUF, net ultrafiltration; RRT, renal replacement therapy; SOFA, Sequential Organ Failure Assessment.

^a^ Daily and cumulative fluid balances were calculated during continuous venovenous hemodiafiltration after excluding the NUF volume from the output volume calculation.

### Association of NUF Rate With Outcomes

Patients with NUF rates greater than 1.75 mL/kg/h had a longer median (IQR) duration of mechanical ventilation (high: 7 [3-12] days; middle: 6 [2-12] days; low: 3 [1-7] days; *P* < .001), ICU length of stay (high: 9 [5-16] days; middle: 8 [4-15] days; low: 5 [2-10] days; *P* < .001), and hospital length of stay (high: 10 [6-16] days; middle: 9 [5-16] days; low: 6 [3-11] days; *P* < .001) compared with patients in the lowest and middle tertile (Table 2). A greater proportion of patients receiving NUF greater than 1.75 mL/kg/h were dependent on dialysis by day 28 (high: 64 of 292 surviving patients [21.9%]; middle: 37 of 317 surviving patients [11.7%]; low: 19 of 290 surviving patients [6.6%]; *P* < .001); however, there was no difference by day 90 (high: 17 of 246 surviving patients [6.9%]; middle: 17 of 291 surviving patients [5.8%]; low: 10 of 263 surviving patients [3.8%]; *P* = .28). This was primarily owing to higher mortality among patients receiving NUFgreater than 1.75 mL/kg/h (high: 232 patients [48.6%]; middle: 188 patients [39.2%]; low: 214 patients [44.9%]; *P* = .01) (eFigure 7 in the Supplement).

Compared with NUF rates less than 1.01 mL/kg/h, NUF rates greater than 1.75 mL/kg/h were associated with lower survival, which was variable and yet persisted from day 7 to day 90. During this period, death occurred in 51 patients (14.7%) treated with NUF greater than 1.75 mL/kg/h compared with 30 patients (8.6%) treated with NUF less than 1.01 mL/kg/h from day 7 to 12 (aHR, 1.51; 95% CI, 1.13-2.02); 45 patients (15.3%) treated with NUF greater than 1.75 mL/kg/h compared with 25 patients (7.9%) treated with NUF less than 1.01 mL/kg/h from day 13 to day 26 (aHR, 1.52; 95% CI, 1.11-2.07); and 48 patients (19.2%) treated with NUF greater than 1.75 mL/kg/h compared with 29 patients (9.9%) treated with NUF less than 1.01 mL/kg/h from day 27 to 90 (aHR, 1.66; 95% CI, 1.16-2.39) (Table 3; Figure) (eTable 1 and eTable 4 in the Supplement). This association was not attenuated by organ edema strata, sepsis, eGFR less than 60 mL/in/1.73 m^2^, mean cardiovascular SOFA score of 3 or higher, or high-intensity CVVHDF (eTable 8 in the Supplement).

**Table 3.** Association of NUF With Survival From Gray Model

NUF Rate	Model	Hazard Ratio (95% CI)^a^					*P* Value
		0-2 d	3-6 d	7-12 d	13-26 d	27-90 d	
No. of patients at risk		1390	1216	1085	976	862	
>1.75 mL/kg/h vs <1.01 mL/kg/h	Unadjusted	0.62 (0.47-0.82)	0.86 (0.67-1.10)	1.31 (1.02-1.68)	1.46 (1.11-1.91)	1.70 (1.23-2.34)	<.001
	Adjusted^b^	1.13 (0.81-1.57)	1.24 (0.93-1.66)	1.51 (1.13-2.02)	1.52 (1.11-2.07)	1.66 (1.16-2.39)	.01
>1.75 mL/kg/h vs 1.01-1.75 mL/kg/h	Unadjusted	0.97 (0.72-1.30)	1.16 (0.89-1.49)	1.49 (1.16-1.91)	1.40 (1.07-1.82)	1.66 (1.21-2.28)	.002
	Adjusted^b^	1.12 (0.81-1.56)	1.18 (0.89-1.57)	1.44 (1.10-1.90)	1.42 (1.07-1.89)	1.77 (1.26-2.49)	.004
1.01-1.75 mL/kg/h vs <1.01 mL/kg/h	Unadjusted	0.64 (0.48-0.85)	0.74 (0.57-0.96)	0.84 (0.64-1.09)	1.14 (0.86-1.52)	0.97 (0.69-1.37)	.006
	Adjusted^b^	1.01 (0.74-1.39)	1.09 (0.82-1.46)	1.00 (0.74-1.34)	1.15 (0.84-1.52)	0.85 (0.58-1.25)	.59
NUF per 0.50-mL/kg/h increase	Unadjusted	0.90 (0.85-0.97)	0.97 (0.92-1.03)	1.06 (1.00-1.12)	1.09 (1.03-1.16)	1.11 (1.04-1.19)	<.001
	Adjusted^b^	1.03 (0.97-1.09)	1.05 (1.00-1.11)	1.08 (1.02-1.15)	1.11 (1.04-1.18)	1.13 (1.05-1.22)	.003

Abbreviation: NUF, net ultrafiltration.

^a^ Unadjusted and adjusted hazard ratios estimated from Gray model for association of NUF rate with mortality for each time interval are shown. Models included 5 intervals and 4 nodes, with the default timing of nodes chosen by the statistical program based on number of events within each interval. A hazard ratio less than 1 suggests that the NUF rate is associated with lower mortality, and a hazard ratio greater than 1 suggests that the NUF rate is associated with higher mortality within each time interval. *P* values reported are for the ranges of hazard ratios across time intervals from the model.

^b^ Adjusted for age category; female sex; premorbid estimated glomerular filtration rate; duration from intensive care unit admission to study enrollment; Acute Physiology and Chronic Health Evaluation III category in the 24 hours prior to study enrollment; total Sequential Organ Failure Assessment score at study enrollment; presence of organ edema, sepsis, and use of mechanical ventilation at enrollment; daily mean cardiovascular Sequential Organ Failure Assessment score during treatment with continuous venovenous hemodiafiltration; cumulative daily fluid balance from study enrollment to intensive care unit discharge; duration of renal replacement therapy; source of admission, including whether the patient was transferred to the intensive care unit from an emergency department, hospital ward, operating room after elective or emergency surgery, another hospital or intensive care unit; hospital type; and hospital region. Models were fitted in 1341 patients with complete data.

**Figure.**
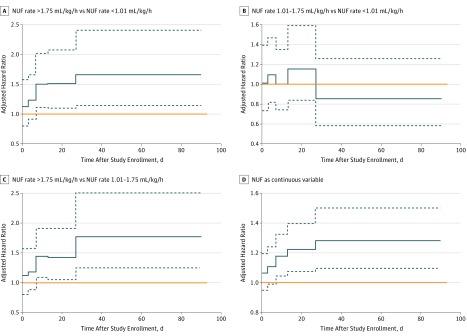
Net Ultrafiltration (NUF) Rate and Survival From Gray Model Hazard ratios (blue solid lines) are shown with 95% CIs (blue dotted lines). The orange line indicates a hazard ratio of 1. A hazard ratio less than 1 suggests that the NUF rate is associated with lower mortality, and a hazard ratio greater than 1 suggests that the NUF is associated with higher mortality. A, The risk of death associated with an NUFrate greater than 1.75 mL/kg/h compared with an NUFrate slower than 1.01 mL/kg/h was 51% for day 7 to 12, 52% for day 13 to 26, and 66% for day 27 to 90. B, An NUFrate from 1.01 to 1.75 mL/kg/h was not associated with death. C, For an NUFrate greater than 1.75 mL/kg/h compared with an NUFrate from 1.01 to 1.75 mL/kg/h, the risk of death was 44% for day 7 to 12, 42% for day 13 to 26, and 77% for day 27 to 90. D, Every 0.50-mL/kg/h increase in NUFrate was associated with death: 5% for day 3 to 6, 8% for day 7 to 12, 11% for day 13 to 26, and 13% for day 27 to 90.

A similar hazard was present for patients treated with NUF greater than 1.75 mL/kg/h compared with patients treated with an NUF rate from 1.01 to 1.75 mL/kg/h from day 7 to 90. During this period, 28 patients (7.1%) in the middle NUF rate group died from day 7 to 12 (aHR, 1.44; 95% CI, 1.10-1.90); 44 patients (12.1%) died from day 13 to 26 (aHR, 1.42; 95% CI, 1.07-1.89); and 29 patients (9.1%) died from day 27 to 90 (aHR, 1.77; 95% CI, 1.26-2.49) (Figure).

Every 0.5-mL/kg/h increase in NUF rate was associated with increased mortality from day 3 to day 90 (days 3-6: aHR, 1.05; 95% CI, 1.00-1.11; days 7-12: aHR, 1.08; 95% CI, 1.02-1.15; days 13-26: aHR, 1.11; 95% CI, 1.04-1.18; days 27-90: aHR, 1.13; 95% CI, 1.05-1.22) (Figure). Of patients with available premorbid creatinine levels, NUF rates greater than 1.75 mL/kg/h were also associated with increased mortality (days 16-33; aHR, 1.75; 95% CI, 1.15-2.67; *P* = .02). Net ultrafiltration rate was significantly associated with risk of death in the presence of cumulative fluid balance (aHR, 1.00; 95% CI, 1.00-1.00; *P *for interaction = .001). Using a joint model, longitudinal increase in NUF rate was associated with risk of death (β = .056; *P* < .001).

### Sensitivity and Subgroup Analyses

In the propensity score–matched cohort (405 matched pairs), an NUFrate greater than 1.75 mL/kg/h compared with 1.75 mL/kg/h or less was associated with mortality (192 patients [47.5%] vs 164 patients [40.5%]; *P* = .04; unadjusted odds ratio [OR], 1.33; 95% CI, 1.01-1.76; *P* = .04) (eTable 3 and eFigure 4 in the Supplement). An NUF rate greater than 1.75 mL/kg/h was associated with reduced survival using a lower threshold of 0.05 mL/kg/h (aHR, 1.62; 95% CI, 1.12-2.34) and a higher threshold of 0.05 mL/kg/h (aHR, 1.68; 95% CI, 1.16-2.43) (eTable 5 in the Supplement). Restricting to 72 hours of CVVHDF, NUF greater than 1.65 mL/kg/h compared with NUF less than 0.82 mL/kg/h was associated with increased mortality (aHR, 1.72; 95% CI, 1.20-2.47). Using maximum values, an NUF rate greater than 2.66 mL/kg/h compared with an NUF rate less than 1.57 mL/kg/h was associated with death (aHR, 1.95; 95% CI, 1.44-2.65).

After excluding 92 patients with NUF rates less than 0.01 mL/kg/h, NUF rates greater than 1.75 mL/kg/h were associated with lower survival (aHR, 1.57; 95% CI, 1.08-2.28). A similar hazard persisted after including the 31 patients with missing treatment hours and assigning them an NUF rate of 0 mL/kg/h (aHR, 1.60; 95% CI, 1.12-2.29) as well as assigning them an NUF rate of 1.43 mL/kg/h (aHR, 1.65; 95% CI, 1.15-2.38). The association persisted when the nodes in the Gray model were increased (aHR, 1.68; 95% CI, 1.16-2.44) or decreased (aHR, 1.63; 95% CI, 1.14-2.33).

An NUF rate greater than 1.75 mL/kg/h was associated with lower survival after adjusting for SOFA scores (aHR, 1.64; 95% CI, 1.14-2.38), use of blood products and protein supplementation (aHR, 1.60; 95% CI, 1.14-2.25), cumulative fluid balance including NUF volume (aHR, 1.74; 95% CI, 1.21-2.51), and after excluding cumulative fluid balance (aHR, 1.72; 95% CI, 1.20-2.47).

Using stratified analysis, CVVDHF for 3 or more days and CVVDHF for 5 or more days were associated with death (≥3 days: aHR, 1.99; 95% CI, 1.22-3.23; ≥5 days: aHR, 1.93; 95% CI, 1.00-3.75). Of patients with negative fluid balance, the cutoff values were NUF rates less than 1.39 mL/kg/h, from 1.39 to 2.03 mL/kg/h, and greater than 2.03 mL/kg/h. Compared with NUF rates less than 1.39 mL/kg/h, NUF rates greater than 2.03 mL/kg/h were associated with death (aHR, 2.71; 95% CI, 1.56-4.69). Using logistic regression, an NUF rate greater than 1.75 mL/kg/h was not associated with death compared with an NUF rate less than 1.01 mL/kg/h (adjusted OR, 1.25; 95% CI, 0.91-1.73) (eTable 6 in the Supplement). However, every 0.5-mL/kg/h increase was associated with a 7% increase in odds of death (adjusted OR, 1.07; 95% CI, 1.00-1.15) (eTable 7 and eFigure 6 in the Supplement).

Using subgroup analyses, an NUFrate greater than 1.75 mL/kg/h was associated with mortality among patients with and without organ edema (with organ edema: aHR, 1.61; 95% CI, 1.01-2.55; without organ edema: aHR, 1.75; 95% CI, 1.06-2.88); with and without sepsis (with sepsis: aHR, 2.19; 95% CI, 1.26-3.80; without sepsis: aHR, 1.72; 95% CI, 1.14-2.59); and with eGFR greater than 60 mL/min/1.73 m^2^ (aHR, 2.30; 95% CI, 1.21-4.38). Among 759 patients (52.9%) with eGFR less than 60 mL/min/1.73 m^2^, the cutoff values were NUF rates less than 0.95 mL/kg/h, from 0.95 to 1.71 mL/kg/h, and greater than 1.71 mL/kg/h. Compared with an NUFrate less than 0.95 mL/kg/h, an NUFrate greater than 1.71 mL/kg/h was associated with death (aHR, 1.53; 95% CI, 1.04-2.26). An NUFrate greater than 1.75 mL/kg/h was associated with mortality among patients with cardiovascular SOFA scores of 3 or greater (aHR, 1.89; 95% CI, 1.27-2.81) and high-intensity CVVHDF (aHR, 2.22; 95% CI, 1.35-3.66) (eTable 8 in the Supplement).

### Complications and Adverse Events

A greater proportion of patients receiving NUF greater than 1.75 mL/kg/h developed hypophosphatemia compared with patients receiving NUF from 1.01 to 1.75 mL/kg/h and less than 1.01 mL/kg/h (high: 308 of 477 patients at risk [64.6%]; middle: 293 of 472 patients at risk [62.1%]; low: 247 of 466 patients at risk [53.0%]; *P* < .001) (Table 4). The frequency of hypophosphatemic episodes was also high (high: 1003 episodes; middle: 893 episodes; low: 627 episodes; *P* < .001). However, when adjusted for differences in effluent flow and duration of CVVHDF, an NUF rate greater than 1.75 mL/kg/h was not associated with risk of hypophosphatemia (adjusted OR, 1.02; 95% CI, 0.76-1.36; *P* = .89). More patients with NUF rates greater than 1.75 mL/kg/h developed cardiac arrhythmias requiring treatment and had an increased number of these episodes, but the associations were not significant (patients developing arrhythmia requiring treatment: high: 176 of 478 patients at risk [36.8%]; middle: 175 of 479 patients at risk [36.5%]; low: 147 of 477 patients at risk [30.8%]; *P* = .08; number of episodes: high: 286 episodes; middle: 264 episodes; low: 237 episodes; *P* = .08).

**Table 4.** Summary of Complications by NUF Rate

Complication	NUF Rate <1.01 mL/kg/h	NUF Rate 1.01-1.75 mL/kg/h	NUF Rate >1.75 mL/kg/h	*P* Value
Total No.	477	479	478	NA
Hypophosphatemia^a^				
No. of patients/No. at risk (%)	247/466 (53.0)	293/472 (62.1)	308/477 (64.6)	<.001
No. of episodes	627	893	1003	<.001
Hypokalemia^a^				
No. of patients/No. at risk (%)	116/472 (24.6)	116/477 (24.3)	109/477 (22.8)	.79
No. of episodes	199	179	206	.39
Arrhythmia				
No. of patients/No. at risk (%)	191/477 (40.0)	212/479 (44.3)	222/478 (46.4)	.13
No. of episodes	340	386	410	.06
Arrhythmia requiring treatment				
No. of patients/No. at risk (%)	147/477 (30.8)	175/479 (36.5)	176/478 (36.8)	.08
No. of episodes	237	264	286	.08
Arrhythmia causing hemodynamic instability				
No. of patients/No. at risk (%)	110/477 (23.1)	126/479 (26.3)	133/478 (27.8)	.23
No. of episodes	144	179	217	.13
Disequilibrium				
No. of patients/No. at risk (%)	2/477 (0.4)	1/479 (0.2)	0/478	.37
No. of episodes	2	1	0	NA
≥1 Serious adverse events				
No. of patients/No. at risk (%)	2/477 (0.4)	5/479 (1.0)	2/478 (0.4)	.37
No. of episodes	2	5	2	NA

Abbreviations: NA, not applicable; NUF, net ultrafiltration.

^a^ Levels were measured in routine morning blood samples.

## Discussion

Among critically ill patients receiving CVVHDF, we found that an NUF rate greater than 1.75 mL/kg/h compared with an NUF rate less than 1.01 mL/kg/h was associated with lower risk-adjusted 90-day survival between day 7 and day 90. These findings are aligned with several recent studies in outpatients with end-stage renal disease^10,11,12,13^ that found that higher NUF rates are associated with decreased survival.

Our findings have several implications. First, the attributable risk associated with an NUF rate greater than 1.75 mL/kg/h was significantly higher (aHR, 1.66; 95% CI, 1.16-2.39) than risk associated with cumulative positive fluid balance (aHR, 1.00; 95% CI, 1.00-1.00) (eTable 4 in the Supplement). Moreover, there was an interaction between the NUF rate and cumulative fluid balance that considerably increased this risk, which may explain the high mortality among patients treated with CVVHDF. Notably, this risk was present only after day 7 and is easily modifiable by slowing the NUF rate to less than 1.75 mL/kg/h.

There are many possible biological explanations for late mortality. Decreased circulating volume is associated with decreased coronary perfusion and myocardial ischemia.^7,26^ Repeated ischemia is associated with ventricular remodeling and heart failure.^7^ Gut hypoperfusion is associated with increased permeability, bacterial translocation, and endotoxemia, which is associated with chronic inflammation and cardiac stunning.^27^

Hypotension associated with a high NUF rate may result in administration of fluid with subsequent fluid overload, which is associated with ventricular hypertrophy and fibrosis, predisposing the patient to heart failure and sudden death.^28,29,30^ The propensity toward higher frequency of cardiac arrhythmias in patients with a high NUF rate supported this finding. An NUF rate greater than 1.75 mL/kg/h was also associated with risk of hypophosphatemia, which has also been noted in the 2 different trials of intensity of solute control^14,31^ as well as with increased duration of CVVHDF.^32^ Hypophosphatemia may also predispose to cardiac arrhythmias and other undesirable biological effects.^33,34,35^

Second, our study suggests that a more modest NUF rate less than 1.75 mL/kg/h is associated with lowest risk (eFigure 2 in the Supplement). This finding is consistent with other studies in patients with end-stage renal disease,^13,36,37^ in which lower rates and longer treatment duration were associated with survival. Nevertheless, randomized clinical trials are required to confirm our findings.

Third, while a lower NUF rate might be associated with improved outcomes, it is likely to prolong treatment duration, and this has to be balanced against the need for fluid removal in a critically ill patient. For example, pulmonary edema in a patient with severe heart failure or refractory hypoxemia in a patient with acute respiratory distress syndrome may need a greater NUF rate for a short period of time to prevent sudden death.

In a single-center study, Murugan et al^9^ found that among the subgroup of 487 patients who only received CVVHDF, an NUF rate less than 0.5 mL/kg/h compared with an NUF rate greater than 1 mL/kg/h was associated with higher mortality. Although the reason for the differences between the 2 studies is unclear, it is important to note that there are considerable differences in study design and patient population between them. Nevertheless, these differential findings emphasize the need for randomized clinical trials to examine the relationship of NUF rates with outcomes.

### Limitations

Our study has several limitations. First, findings may be biased by measured and unmeasured confounding at the patient and hospital levels. Nevertheless, the joint model matched propensity score analysis, and the logistic regression provided alternative methods to handle measured confounders and support the primary analysis. Second, data on race/ethnicity, comorbid conditions, and episodes of hypotension during treatment were not measured. Third, there were 31 patients with missing treatment hours; however, including these patients in the analysis did not change the results. Fourth, fluid balance prior to initiation of CVVHDF was unavailable, a limitation that was addressed using the organ edema variable as a surrogate for fluid overload. Given these limitations, the risk associated with an NUF rate greater than 1.75 mL/kg/h is likely to be smaller than measured in this study.

## Conclusions

In this study of critically ill patients receiving CVVHDF, an NUF rate greater than 1.75 mL/kg/h compared with an NUF rate less than 1.01 mL/kg/h was associated with lower survival. Although the study design does not exclude the possibility of residual confounding owing to unmeasured risk factors, a randomized clinical trial is required to validate these findings before they can be applied to clinical practice.

## Notes

Supplement. **eAppendix 1.** The RENAL Cohort **eAppendix 2.** Statistical Method for Imputation of Missing Premorbid Creatinine Values and Estimated Glomerular Filtration Rate **eAppendix 3.** Multivariable Gray Piecewise-Constant Time-Varying Coefficients Regression Model for Time to Mortality **eAppendix 4.** Joint Model **eAppendix 5.** Statistical Methods for Propensity Score Estimation and Matching **eAppendix 6.** Multivariable Logistic Regression Model **eTable 1.** Number of Deaths Within Each Time Interval From Primary Gray Model **eTable 2.** Characteristics of Patients With Missing Treatment Duration During Continuous Venovenous Hemodiafiltration **eTable 3.** Patient Characteristics Before and After Propensity Score Matching **eTable 4.** Association of NUF Rate With Mortality From Primary Gray Model **eTable 5.** Sensitivity Analyses of NUF Rate and Mortality **eTable 6.** Association of NUF Rate With Risk-Adjusted Mortality From Logistic Regression **eTable 7.** Association of NUF Rate With Risk-Adjusted Mortality Using NUF as a Continuous Variable From Logistic Regression **eTable 8.** Subgroup Analyses of NUF Rate and Mortality **eFigure 1.** Distributions of Imputed and Unimputed Premorbid Serum Creatinine Levels and Estimated Glomerular Filtration Rate **eFigure 2.** Association of NUF Rate With Crude 90-Day Mortality **eFigure 3.** Violation of Proportional Hazards Assumptions by NUF Rate Variable **eFigure 4.** Distribution of Propensity Scores for Patients Who Received NUF Rate >1.75 mL/kg/hr Among Matched (N=405) and Unmatched Patients (N=532) **eFigure 5.** Logistic Regression Model Calibration and Discrimination **eFigure 6.** Crude 90-Day Mortality and Predicted Risk of Death by NUF Rate **eFigure 7.** Association of NUF Rate With 90-Day Survival **eReferences**
