# Supplementary material for: Association of Net Ultrafiltration Rate With Mortality Among Critically Ill Adults With Acute Kidney Injury Receiving Continuous Venovenous Hemodiafiltration: A Secondary Analysis of the Randomized Evaluation of Normal vs Augmented Level (RENAL) of Renal Replacement Therapy Trial
Source: JAMA Netw Open. 2019 Jun 7;2(6):e195418. doi: 10.1001/jamanetworkopen.2019.5418 (PMC6563576; doi:10.1001/jamanetworkopen.2019.5418)
Supplement: Supplement. — eAppendix 1. The RENAL Cohort eAppendix 2. Statistical Method for Imputation of Missing Premorbid Creatinine Values and Estimated Glomerular Filtration Rate eAppendix 3. Multivariable Gray Piecewise-Constant Time-Varying Coefficients Regression Model for Time to Mortality eAppendix 4. Joint Model eAppendix 5. Statistical Methods for Propensity Score Estimation and Matching eAppendix 6. Multivariable Logistic Regression Model eTable 1. Number of Deaths Within Each Time Interval From Primary Gray Model eTable 2. Characteristics of Patients With Missing Treatment Duration During Continuous Venovenous Hemodiafiltration eTable 3. Patient Characteristics Before and After Propensity Score Matching eTable 4. Association of NUF Rate With Mortality From Primary Gray Model eTable 5. Sensitivity Analyses of NUF Rate and Mortality eTable 6. Association of NUF Rate With Risk-Adjusted Mortality From Logistic Regression eTable 7. Association of NUF Rate With Risk-Adjusted Mortality Using NUF as a Continuous Variable From Logistic Regression eTable 8. Subgroup Analyses of NUF Rate and Mortality eFigure 1. Distributions of Imputed and Unimputed Premorbid Serum Creatinine Levels and Estimated Glomerular Filtration Rate eFigure 2. Association of NUF Rate With Crude 90-Day Mortality eFigure 3. Violation of Proportional Hazards Assumptions by NUF Rate Variable eFigure 4. Distribution of Propensity Scores for Patients Who Received NUF Rate >1.75 mL/kg/hr Among Matched (N=405) and Unmatched Patients (N=532) eFigure 5. Logistic Regression Model Calibration and Discrimination eFigure 6. Crude 90-Day Mortality and Predicted Risk of Death by NUF Rate eFigure 7. Association of NUF Rate With 90-Day Survival eReferences [file jamanetwopen-2-e195418-s001.pdf]

Supplementary Online Content

Murugan R, Kerti SJ, Chang C-CH, et al. Association of net ultrafiltration rate with mortality among critically ill adults with acute kidney injury receiving continuous venovenous hemodiafiltration: a secondary analysis of the Randomized Evaluation of Normal vs Augmented Level (RENAL) of Renal Replacement Therapy trial. *JAMA Netw Open*. 2019;2(6):e195418. doi:10.1001/jamanetworkopen.2019.5418

eAppendix 1. The RENAL Cohort

eAppendix 2. Statistical Method for Imputation of Missing Premorbid Creatinine Values and Estimated Glomerular Filtration Rate

eAppendix 3. Multivariable Gray Piecewise-Constant Time-Varying Coefficients Regression Model for Time to Mortality

eAppendix 4. Joint Model

eAppendix 5. Statistical Methods for Propensity Score Estimation and Matching

eAppendix 6. Multivariable Logistic Regression Model

eTable 1. Number of Deaths Within Each Time Interval From Primary Gray Model

eTable 2. Characteristics of Patients With Missing Treatment Duration During Continuous Venovenous Hemodiafiltration

eTable 3. Patient Characteristics Before and After Propensity Score Matching

eTable 4. Association of NUF Rate With Mortality From Primary Gray Model

eTable 5. Sensitivity Analyses of NUF Rate and Mortality

eTable 6. Association of NUF Rate With Risk-Adjusted Mortality From Logistic Regression

eTable 7. Association of NUF Rate With Risk-Adjusted Mortality Using NUF as a Continuous Variable From Logistic Regression

eTable 8. Subgroup Analyses of NUF Rate and Mortality

eFigure 1. Distributions of Imputed and Unimputed Premorbid Serum Creatinine and Estimated Glomerular Filtration Rate

eFigure 2. Association of NUF Rate With Crude 90-Day Mortality

eFigure 3. Violation of Proportional Hazards Assumptions by NUF Rate Variable

eFigure 4. Distribution of Propensity Scores for Patients Who Received NUF Rate >1.75 mL/kg/hr Among Matched (N=405) and Unmatched Patients (N=532)

eFigure 5. Logistic Regression Model Calibration and Discrimination

eFigure 6. Crude 90-Day Mortality and Predicted Risk of Death by NUF Rate

eFigure 7. Association of NUF Rate With 90-Day Survival

eReferences

This supplementary material has been provided by the authors to give readers additional information about their work.

© 2019 Murugan R et al. *JAMA Network Open.*

eAppendix 1. The RENAL Cohort

Critically ill patients were eligible for enrollment in the clinical trial if they met all of the following inclusion criteria:[1]

1. The treating clinician believed that the patient required continuous renal replacement therapy (CRRT) for treatment of acute kidney injury.
2. The clinician is uncertain about the balance of benefits and risks likely to be conferred by treatment with higher intensity or lower intensity CRRT.
3. The treating clinicians anticipate treating the patient with CRRT for at least 72 hours.
4. Informed consent has been obtained.
5. The patient fulfils ONE of the following clinical criteria for initiating CRRT:
    - Oliguria (urine output <100ml/6hr) that has been unresponsive to fluid resuscitation measures.
    - Hyperkalemia ([K+] >6.5 mmol/L).
    - Severe acidemia (pH <7.2).
    - Urea >25 mmol/liter.
    - Creatinine >300 µmol/L.
    - Clinically significant organ edema in the setting of acute kidney injury (*e.g.*, pulmonary edema).

Patients were excluded from the study if, in the opinion or knowledge of the responsible clinician, any of the following criteria were present:

1. Patient age is <18 years.
2. Death is imminent (<24 hours).
3. There is a strong likelihood that the study treatment would not be continued in accordance with the study protocol.
4. The patient has been treated with CRRT or other dialysis previously during the same hospital admission.
5. The patient was on maintenance dialysis prior to the current hospitalization.
6. The patient’s body weight is <60 kg or >100 kg.
7. Any other major illness that, in the investigator’s judgment substantially increased the risk associated with the subject’s participation in this study.

Study treatment (CRRT) was discontinued if or when any of the following criteria were met:

1. Patient withdraws consent for study treatment, or
2. Patient dies, or
3. Patient leaves the ICU, or

© 2019 Murugan R et al. *JAMA Network Open.*

4. Patient urine output is >400ml in the previous 24 hours and the treating clinicians consider renal function has recovered to the point that further renal replacement therapy is not needed, or
5. The treating clinicians consider it is the patient's best interest to change from CRRT to intermittent dialysis. If this occurred after less than 72 hours of CRRT the patient was classified as a screen failure and was not enrolled in the trial.

Of 1,508 patients enrolled in the RENAL trial, consent was withdrawn in 43 patients. Of the remaining 1,465 eligible patients, the outcome data was not available in 1 patient. Patients (n=1,464) were randomly assigned to continuous venovenous hemodiafiltration (CVVHDF) with effluent flow at 40 mL/kg/hr (higher intensity) or 25 mL/kg/hr (lower intensity) for solute clearance. Replacement fluid was delivered into the extracorporeal circuit after the filter (*i.e.,* postdilution) with a ratio of dialysate to replacement fluid of 1:1. Blood flow was kept above 150 mL per minute. Filters with the AN69 membrane (Gambro) were used. Hemosol BO fluid (Gambro) was used as the dialysate and replacement fluid.

The presence of clinically significant organ edema was determined by clinicians based on radiologically confirmed pulmonary edema or visible or palpable organ edema (cardiac or gastrointestinal) at surgery. Incomplete study days (day of enrollment and day of discharge or death) were considered full data collection days.[2] Study treatment was discontinued on death, discharge from ICU, or recovery of renal function. The primary end-point was time-to-mortality censored at 90 days after study randomization. The RENAL trial found no difference in 90 day mortality between the two intensities of renal replacement therapy.[1]

© 2019 Murugan R et al. *JAMA Network Open.*

eAppendix 2. Statistical Method for Imputation of Missing Premorbid Creatinine Values and Estimated Glomerular Filtration Rate

Premorbid renal function as determined by serum creatinine and estimated glomerular filtration rate (eGFR) is a strong predictor of mortality. However, premorbid serum creatinine is frequently unavailable in acute kidney injury studies due to missing data. Generally, there are three different patterns of missing data.[3,4] First, data may be missing *completely at random* when the probability of missing values does not depend on any observed or unobserved variables. Second, data may be missing *not completely at random* if the probability of missing values depends on observed variables. Third, data may be missing non-random when the probability of missing values depends unobserved variables. Because the decision to measure premorbid serum creatinine is usually based on some existing clinical information, missing premorbid creatinine data are generally considered missing *not completely at random*.[5,6]

Multiple imputation is a widely used approach to estimate premorbid creatinine values when data are missing at random.[5] We used the multivariable imputation by chained equation (MICE) method[7] to impute the creatinine values using age, sex, and weight as predictors among 637 subjects (44.4%) with missing premorbid serum creatinine values. By leveraging known patient characteristics and accounting for uncertainty in the multiple estimations of missing values, multiple imputation preserves sample size and reduces bias while examining association between variables.[4] For each multiple imputation strategy, values of premorbid serum creatinine were imputed using linear regression. Serum creatinine was imputed 20 times using MICE[7] and values were averaged to obtain the final estimate.

We subsequently used the Modification of Diet in Renal Disease (MDRD) equation to estimate the premorbid glomerular filtration rate using the imputed and unimputed creatinine.[8] eFigure 1 shows the distribution of imputed and unimputed premorbid serum creatinine and eGFR. The distributions were compared using the Kolmogorov-Smirnov Two-Sample test, which is a nonparametric test of the equality of continuous, one-dimensional probability distributions that can be used to compare two samples. We found no difference in distribution of imputed and unimputed premorbid creatinine values (P=0.29) and corresponding premorbid eGFR (P=0.13; eFigure 1).

© 2019 Murugan R et al. *JAMA Network Open.*

eAppendix 3. Multivariable Gray Piecewise-Constant Time-Varying Coefficients Regression Model for Time to Mortality

There are well known risk factors for death after fluid overload in critically ill patients such as age, premorbid renal function, shock, severity of illness or cumulative fluid overload. The impact of these factors on mortality change overtime. For instance, an elderly patient in shock is initially at greater risk of death from shock than from premorbid renal function or cumulative fluid overload. Should she survive shock, premorbid renal function and cumulative fluid overload will become important determinants of late survival.[9,10] This observation could be important when using survival models to estimate differences in outcomes between different groups (*e.g.*, NUF rate).

Survival models analyze time-to-event data to identify predictors of outcome. The most common approach, described by Cox,[11] assumes constant hazard ratios throughout a subject's time course. By assuming constant hazard ratios for all risk factors, Cox models assume the relative contribution of each risk factor to mortality is constant over time. This assumption is known as the "proportional hazards" and does not hold true in patients with fluid overload,[9] nor in other acute illness diseases (*e.g.*, sepsis).[12]

Considering again our example, the hazard ratio for shock at presentation will likely decrease over time because, if the subject survives the initial shock, the fact that shock was present will become less and less important as time passes. Consequently, traditional survival models, which depend on proportional hazards such as the Cox model, do not adequately describe survival after fluid overload in critically ill patients.[9,10]

Gray's piecewise-constant time-varying coefficients survival model[13-16] is an approach that directly estimates how the hazards from individual risk factors change over time and therefore is better suited for modeling survival after fluid overload.[9] We fitted Gray's model for three reasons: First, NUF variable violated proportionality assumption (eFigure 3).[11] Second, the Gray's model does not assume time-invariant effects of the exposure.[15] This is important because the association between NUF rate and mortality is likely to vary over time.[9,10] Third, the Gray's model accounts for differences in exposure time, which is important because patients with low rate of NUF and positive fluid balance will have shorter survival time within each time interval.[10]

The advantage of Gray's model is that it does not rely on the proportional hazards assumption, does not require arbitrary decisions regarding time intervals as it assigns the duration of these time intervals automatically, based on the actual data themselves, and provides a richer description of risk of death over-time.[15] Another advantage of the

© 2019 Murugan R et al. *JAMA Network Open.*

Gray's model is that it provides coefficients that can be interpreted in the same way as those obtained from a traditional Cox model and provides a statistical method sensitive to clinical nuance. For example, the model demonstrated that the association between NUF rate >1.75 mL/kg/hr compared with <1.01 mL/kg/hr and mortality was variable: there was no association between NUF rate >1.75 mL/kg/hr and mortality until day 6, however the risk increased from day 7 to 26 and then stayed constant from day 27 to day 90 (Figure A in the manuscript).

© 2019 Murugan R et al. *JAMA Network Open.*

eAppendix 4. Joint Model

Longitudinal data and time-to-event data can be modelled separately using linear mixed effects model and Cox proportional hazards model. However, these separate models fail to account for correlation between the longitudinal data and the time-to-event data that may result in biased estimates of treatment effect. A Joint model accounts for this correlation by simultaneously modelling the time-to-event and longitudinal data in a single unified model.[17,18]

We conducted longitudinal analyses using Joint model to account for correlation between daily NUF rate and cardiovascular (CV) SOFA score over time and its association with survival. We chose Joint modelling because it allowed us to simultaneously model associations between NUF rate, changes in NUF rate and CV SOFA score over time, NUF rate and CV SOFA before death and risk of time-to-death in a single unified model. The effect on the hazard of a longitudinally measured covariate is accomplished by assuming that the hazard is a function of the “true” but latent trajectory that defines the longitudinal profile.

This approach is similar to the time dependent model in that it uses the current value of the time dependent covariate as the major driving force affecting time-to-death, but the use of a linear model for the longitudinal portion allows a more precise estimate of the NUF rate and CV SOFA score before death. The parameter estimates(β) for the baseline NUF rate and the change in NUF rate immediately prior to death adjusting for daily CV SOFA score can be used to estimate the effect of these variables on risk of death. If we transform the parameter estimate as 100*(exp(β*K)-1), this will give the percent increase or decrease in the hazard per K unit increase in NUF rate. However, the association estimates derived for rate of change in NUF rate over time cannot be transformed in the same manner to the magnitude of the hazard.

Using Joint modelling, we fitted daily NUF rates and CV SOFA score as longitudinal variables and other baseline covariates in the Cox model. We adjusted for age; sex; eGFR; duration from ICU admission to study enrollment; APACHE-III score; baseline total SOFA score; organ edema, sepsis and use of mechanical ventilation; cumulative daily fluid balance; duration of CVVHDF; source of admission including whether the patient was transferred to the ICU from an emergency department, hospital ward, operating room after elective or emergency surgery, another hospital or ICU; hospital type; and hospital region. The association parameter was 0.056 and the P value was significant at <0.001. A parameter value of <0 implies that increases in the longitudinal NUF rate after adjusting for CV SOFA score and other baseline covariates decreases time-to-death (or increased hazard of death). Table below shows the interpretation of the coefficients from a Joint model and its relationship to time-to-event.

Value of β	Longitudinal Variables	Time-to-Death	Hazard
Less than 0	Increase	Increase	Decrease
Greater than 0	Increase	Decrease	Increase

© 2019 Murugan R et al. *JAMA Network Open.*

eAppendix 5. Statistical Methods for Propensity Score Estimation and Matching

We generated a propensity score (logit) using logistic regression and then matched 405 patients out of the possible 478 patients who received NUF rate >1.75 mL/kg/hr on a 1:1 basis with 405 patients of the 956 patients who received NUF rate ≤1.75 mL/kg/hr, on a random seed, nearest neighbour, without replacement and a caliper distance of 0.05 (approximately 5% of the standard deviation of the logits) (eFigure 4; eTable 3).

The starting model included all available patient characteristics to maximize patient similarity across the two groups. Variables were removed from the model if their standard errors were greater than 0.1 to ensure that the model was not over-fit. Model calibration and discrimination were not calculated because the goal was to match the patients as closely as possible with the NUF rate >1.75 mL/kg/hr group instead of predicting NUF rate >1.75 mL/kg/hr group. An odds ratio for 90 day mortality was generated for NUF rate >1.75 mL/kg/hr group using an unadjusted logistic regression model restricted to 405 propensity-matched subjects (total N=810). Propensity score generation and matching were run using SAS 9.4 (SAS Institute, Cary, NC, USA).

© 2019 Murugan R et al. *JAMA Network Open.*

eAppendix 6. Multivariable Logistic Regression Model

Univariable and multivariable modelling of the association between NUF rate and 90-day mortality was performed using logistic regression adjusted for covariates with robust standard errors and mixed effects to account for nonindependence of cases across ICUs. Binary covariates were modelled as indicator covariates and continuous variables included as linear covariates after assessing for nonlinear relationships. Variance Inflation Factor (VIF) were generated to confirm that there was no collinearity among the independent variables. Specifically, the VIF for the NUF variable was 1.12 and cumulative fluid balance variable was 1.41. Using these covariates, a multivariable logistic regression analysis for 90-day mortality had adequate calibration (Hosmer-Lemeshow goodness-of-fit test for 10 groups, P=0.18; eFigure 5A) and discrimination (area under the receiver operating characteristic curve, 0.78; eFigure 5B) in internal validation. To predict the risk of death across a range of NUF rates, we restricted the cohort to NUF rate of ≤5 mL/kg/hr (n=1,428; 99.6%). We then used predictive margins adjusted for covariates to predict risk of death by NUF rate (eFigure 6).

© 2019 Murugan R et al. *JAMA Network Open.*

eTable 1. Number of Deaths Within Each Time Interval From Primary Gray Model

Characteristic	0 – 2 days	3 – 6 days	7 – 12 days	13 – 26 days	27 – 90 days
<1.01 mL/kg/hr					
No. of deaths/No. at risk (%)	82/477 (17.2)	48/395 (12.2)	30/347 (8.6)	25/317 (7.9)	29/292 (9.9)
1.01 – 1.75 mL/kg/hr					
No. of deaths/No. at risk (%)	48/479 (10.0)	39/431 (9.0)	28/392 (7.1)	44/364 (12.1)	29/320 (9.1)
>1.75 mL/kg/hr					
No. of deaths/No. at risk (%)	44/434 (10.1)	44/390 (11.3)	51/346 (14.7)	45/295 (15.3)	48/250 (19.2)
Total No. of deaths/No. at risk	174/1390 (12.5)	131/1216 (11)	109/1085 (10)	114/976 (11.6)	106/862 (12.3)

© 2019 Murugan R et al. *JAMA Network Open.*

eTable 2. Characteristics of Patients With Missing Treatment Duration During Continuous Venovenous Hemodiafiltration

Characteristic	Study Cohort (N=1,434)	Excluded Cohort (N=31)	P Value
Age, years, median(IQR)	67.3 (56.9 – 76.3)	66.0 (46 – 73.9)	0.23
Male sex	924 (64.4)	22 (71)	0.45
Weight, kg, median(IQR)	80 (70 – 90)	78 (65 – 90)	0.31
Preadmission eGFR, mL/min/1.73m^2, median(IQR)	53 (32.6 – 73.9)	61.7 (53 – 77.3)	0.05
Time in ICU before study enrollment, hours, median(IQR)	20 (6 – 51)	6.5 (2 – 27)	0.006
Mechanical ventilation	1057 (73.7)	25 (83.3)	0.23
Severe Sepsis	709 (49.4)	14 (46.7)	0.76
APACHE-III score, median(IQR)	100 (84 – 118)	112 (96 – 129)	0.02
Total SOFA score, median(IQR)	8 (6 – 9)	9 (7 – 10)	0.03
Baseline individual SOFA score, median(IQR)			
Cardiovascular	4 (1 – 4)	4 (3 – 4)	0.20
Respiratory	3 (2 – 3)	3 (3 – 4)	0.08
Coagulation	0 (0 – 2)	1 (0 – 2)	0.06
Liver	0 (0 – 2)	1 (0 – 2)	0.41
Source of admission			
Emergency department	341 (25.4)	7 (25.9)	0.23
Hospital ward	378 (28.1)	9 (33.3)	
Another ICU	109 (8.1)	2 (7.4)	
Another hospital	149 (11.1)	5 (18.5)	
OR after emergency surgery	204 (15.2)	2 (7.4)	
OR after elective surgery	162 (12.1)	2 (7.2)	
Type of hospital			
1	1023 (71.3)	22 (71)	0.91
2	338 (23.6)	7 (22.6)	
3	23 (1.6)	1 (3.2)	
4	50 (3.5)	1 (3.2)	
Country			
1	104 (7.3)	3 (9.7)	0.61
2	1330 (92.8)	28 (90.3)	
Region			
0	104 (7.3)	3 (9.7)	0.92
1	523 (36.5)	8 (25.8)	
2	85 (5.9)	1 (3.2)	

© 2019 Murugan R et al. *JAMA Network Open.*

3	578 (40.3)	17 (54.8)	

Characteristic	Study Cohort (N=1434)	Excluded Cohort (N=31)	P Value
4	65 (4.5)	0 (0)	
5	18 (1.3)	1 (3.2)	
6	6 (0.4)	0 (0)	
7	55 (3.8)	1 (3.2)	
Duration of mechanical ventilation, days, median(IQR)	5 (2 – 10)	2 (1 – 5)	0.009
ICU length of stay, days, median(IQR)	7 (3 – 14)	2 (1 – 9)	<0.001
Hospital length of stay, days, median(IQR)	8 (5 – 15)	4 (2 – 10)	<0.001
No. of days of RRT, days, median(IQR)	5 (3 – 11)	0 (0 – 1)	<0.001
RRT dependence among survivors			
By day 28	120 (13.4)	1 (7.7)	0.55
By day 90	44 (5.5)	1 (9.1)	0.61
Death			
By day 28	534 (37.2)	18 (56.1)	0.02
By day 90	634 (44.2)	20 (64.5)	0.02

© 2019 Murugan R et al. *JAMA Network Open.*

eTable 3. Patient Characteristics Before and After Propensity Score Matching

Variable	Matching	>1.75 mL/kg/hr	≤1.75 mL/kg/hr	Standardized Bias (%)[a]	Percent Reduction in Bias	P Value
Age, years	Before	63.8 (51.4 – 74.2)	68.7 (59 – 77)	27		<0.001
	After	65.4 (54.4 – 75.2)	64.8 (52.4 – 74.8)	5.7	78.8	0.36
Male sex, No.(%)	Before	283 (59.2)	641 (67)	16		0.003
	After	253 (62.5)	260 (64.2)	3.6	77.5	0.61
eGFR, mL/min/1.72m2	Before	59 (33.3 – 80.2)	49.8 (32.2 – 71.5)	17.8		0.007
	After	50.3 (33.5 – 72.1)	50.3 (33.5 – 72.1)	7.8	56.2	0.41
Time from ICU admission to study enrollment, hours	Before	26 (8 – 63)	17 (4 – 46.5)	11.6		<0.001
	After	24 (8 – 58)	20 (5 – 51)	1.6	86.2	0.04
Total SOFA score	Before	8 (6 – 10)	7 (5 – 9)	18.7		0.002
	After	8 (6 – 10)	8 (6 – 9)	1.1	94.1	0.99
Daily mean cardiovascular SOFA	Before	2.7 (1.3 – 3.7)	2.8 (1.5 – 3.7)	6.0		0.21
	After	2.7 (1.4 – 3.7)	2.5 (1.3 – 3.7)	5.4	10	0.45
APACHE III score	Before	101 (84 – 117)	100 (84 – 118)	1.4		0.92
	After	101 (84 – 117)	99 (83 – 118)	0.8	42.8	0.85
Mechanical ventilation No. (%)	Before	371 (77.6)	686 (71.8)	14		0.02
	After	309 (76.3)	307 (75.8)	1.2	91.4	0.86
Sepsis, No. (%)	Before	253 (53)	456 (47.7)	10.5		0.06
	After	205 (50.6)	201 (49.6)	2.0	81.0	0.77
Organ edema, No.(%)	Before	255 (53.3)	379 (39.6)	27.4		<0.001
	After	200 (49.4)	209 (51.6)	4.4	84.0	0.53
Duration of study treatment, days, median(IQR)	Before	5 (2-10)	3 (2 - 6)	25.3		<0.001
	After	5 (2-9)	4 (2 - 8)	6.9	72.7	0.04
Cumulative fluid balance, L, median(IQR)[b]	Before	11.1 (5.1 - 22.4)	6.1 (2.2 – 11.8)	35.7		<0.001
	After	10.2 (4.6 -20.8)	7.1 (2.5 -14.2)	15.4	56.8	<0.001
Hospital Type, No. (%)						
1	Before	372 (77.8)	651 (68.1)	23.4		<0.001

© 2019 Murugan R et al. *JAMA Network Open.*

Variable	Matching	>1.75 mL/kg/hr	≤1.75 mL/kg/hr	Standardized Bias (%)[a]	Percent Reduction in Bias	P Value
2	Before	95 (19.8)	243 (25.4)	13.9		
3	Before	0	23 (2.4)	-		
4	Before	11 (2.3)	39 (4.1)	11.8		
1	After	312 (77)	312 (77)	0.00	100	0.89
2	After	82 (20.2)	84 (20.7)	1.20	91.4	
3	After	-	-	-	-	
4	After	11 (2.7)	9 (2.2)	3.0	74.5	
Hospital Region, No. (%)						
0	Before	21 (4.4)	83 (8.7)	20.9		
1	Before	197 (41.2)	326 (34.1)	14.4		0.001
2	Before	39 (8.2)	46 (4.8)	12.2		
3	Before	178 (37.2)	400 (41.8)	9.5		
4	Before	22 (4.6)	43 (4.5)	0.5		
5	Before	7 (1.46)	11 (1.1)	2.6		
6	Before	2 (0.4)	4 (0.4)	0.0		
7	Before	12 (2.5)	43 (4.5)	12.7		
0	After	21 (5.2)	14 (3.5)	7.8	62.6	0.82
1	After	162 (40)	166 (41)	2.0	86.1	
2	After	29 (7.2)	30 (7.4)	1.0	91.8	
3	After	156 (38.5)	158 (39)	1.0	89.4	
4	After	19 (4.7)	16 (3.9)	3.5	- 600	
5	After	5 (1.2)	6 (1.5)	2.2	15.4	
6	After	1 (0.2)	4 (1.0)	14.9	-	
7	After	12 (3.0)	11 (2.7)	1.5	88.1	
Source of Admission, No. (%)						
1	Before	85 (19)	256 (28.6)	23.5		<0.001
2	Before	153 (34.2)	225 (25.1)	18.1		
3	Before	40 (8.9)	69 (7.7)	4.2		
4	Before	46 (10.3)	103 (11.5)	3.9		
5	Before	65 (14.5)	139 (15.5)	2.7		
6	Before	58 (13)	104 (11.6)	3.8		
1	After	80 (19.7)	89 (22)	5.6	76.1	0.92
2	After	128 (31.6)	133 (32.8)	2.7	85	

© 2019 Murugan R et al. *JAMA Network Open.*

Variable	Matching	>1.75 mL/kg/hr	≤1.75 mL/kg/hr	Standardized Bias (%)[a]	Percent Reduction in Bias	P Value
3	After	37 (9.1)	37 (9.1)	0.0	100	
4	After	42 (10.4)	37 (9.1)	4.0	- 2.5	
5	After	63 (15.6)	61 (15)	1.4	48.1	
6	After	55 (13.6)	48 (11.8)	5.0	- 31.6	

[a] The standardized bias for continuous covariates was calculated by dividing the difference in means of the covariate between the NUF rate >1.75 mL/kg/hr group and the NUF rate <1.75 mL/kg/hr group by the standard deviation of the NUF rate >1.75 mL/kg/hr group. For categorical variables, the differences in proportions between the NUF rate >1.75 mL/kg/hr group and the NUF rate <1.75 mL/kg/hr group was divided by the standard deviation in the NUF rate >1.75 mL/kg/hr group.

[b] Cumulative fluid balance data were calculated after excluding the NUF volume (exposure variable) from the output fluid calculation.

© 2019 Murugan R et al. *JAMA Network Open.*

eTable 4. Association of NUF Rate With Mortality From Primary Gray Model

Variable	Adjusted Hazard Ratio (95% CI)					
	0 – 2 days	3 – 6 days	7 – 12 days	13 – 26 days	27 – 90 days	P value
<1.01 mL/kg/hr (ref)						
1.01 – 1.75 mL/kg/hr	1.01 (0.74 – 1.38)	1.09 (0.82 – 1.46)	1.00 (0.74 – 1.34)	1.15 (0.84 – 1.58)	0.85 (0.58 – 1.25)	0.59
>1.75 mL/kg/hr	1.13 (0.81 – 1.57)	1.24 (0.93 – 1.66)	1.51 (1.13 – 2.01)	1.52 (1.11 – 2.07)	1.66 (1.15 – 2.39)	0.01
Age , years, <53.25 (ref)						
53.25 – <63.58	0.93 (0.61 – 1.43)	1.15 (0.79 – 1.68)	1.40 (0.96 – 2.04)	1.31 (0.88 – 1.95)	1.78 (1.13 – 2.82)	0.08
63.58 – <71.09	1.18 (0.78 – 1.79)	1.28 (0.87 – 1.89)	1.65 (1.12 – 2.43)	1.61 (1.06 – 2.45)	1.59 (0.98 – 2.58)	0.07
71.09 – <77.72	1.47 (0.96 – 2.24)	1.39 (0.94 – 2.08)	1.30 (0.87 – 1.97)	1.97 (1.30 – 3.01)	1.86 (1.15 – 3.02)	0.01
≥77.72	1.31 (0.86 - 2.00)	1.54 (1.05 – 2.27)	1.59 (1.08 – 2.38)	2.17 (1.43- 3.3)	2.39 (1.48 – 3.88)	0.01
Female sex	1.19 (0.92 – 1.56)	0.94 (0.75 – 1.21)	0.81 (0.63 – 1.04)	0.80 (0.62 – 1.04)	0.91 (0.67 – 1.26)	0.19
Premorbid eGFR	1.00 (1.00 – 1.01)	1.00 (1.00 – 1.01)	1.00 (1.00 – 1.00)	1.00 (1.00 – 1.01)	0.99 (0.99 – 1.00)	0.32
APACHE III score, <82 (ref)						
82 - <95	1.28 (0.81 – 2.03)	1.02 (0.68 – 1.55)	1.02 (0.68 – 1.52)	1.18 (0.79 – 1.77)	1.37 (0.87 – 2.17)	0.54
95 - <107	1.27 (0.80 – 2.02)	1.34 (0.89 – 2.03)	0.99 (0.66 – 1.51)	1.09 (0.71 – 1.66)	1.16 (0.72 – 1.88)	0.59
107 - <122	1.22 (0.78 – 1.94)	1.20 (0.8 – 1.81)	1.62 (1.09 – 2.41)	1.51 (1.00 -2.28)	1.34 (0.82 – 2.19)	0.13
≥122	2.02 (1.31 – 3.13)	1.94 (1.30 – 2.93)	2.02 (1.35 – 3.04)	1.54 (1.00 – 2.38)	1.53 (0.93 – 2.54)	0.01
Sepsis	0.90 (0.68 – 1.2)	0.83 (0.66 – 1.07)	0.87 (0.68 – 1.12)	1.01 (0.78 – 1.32)	1.06 (0.78 – 1.46)	0.62
Mechanical ventilation	1.48 (0.99 – 2.24)	1.36 (0.97 – 1.91)	1.18 (0.85 – 1.64)	1.28 (0.92 – 1.81)	1.57 (1.06 – 2.34)	0.04
Mean total SOFA score at randomization	1.09 (1.03 – 1.16)	1.04 (0.99 – 1.10)	1.05 (1.00 – 1.11)	1.01 (0.96 – 1.07)	0.96 (0.91 – 1.03)	0.01
Mean daily cardiovascular SOFA score	2.35 (2.00 – 2.78)	1.71 (1.5 – 1.95)	1.43 (1.27 – 1.62)	1.39 (1.24 – 1.58)	1.51 (1.00 – 1.32)	0.01
Organ edema	1.08 (0.84 – 1.40)	0.93 (0.74 – 1.18)	1.05 (0.84 – 1.33)	0.93 (0.73 – 1.19)	0.90 (0.67 – 1.22)	0.72
Mean daily fluid balance	0.99 (1.00 – 1.00)	0.99 (1.00 – 1.00)	1.00 (1.00 – 1.00)	1.00 (1.00 – 1.00)	1.00 (1.00 – 1.00)	0.01
Time from ICU admission to study enrollment in hours	1.00 (1.00 – 1.00)	1.00 (1.00 – 1.00)	1.00 (1.00 – 1.00)	1.00 (1.00 – 1.00)	1.00 (1.00 – 1.00)	0.01
Duration of treatment in days	0.74 (0.71 – 0.77)	0.83 (0.81 – 0.86)	0.94 (0.92 – 0.97)	1.00 (0.99 – 1.02)	1.03 (1.02 – 1.04)	0.01
Source of admission (From 1, ref)						

© 2019 Murugan R et al. *JAMA Network Open.*

Variable	Adjusted Hazard Ratio (95% CI)					
	0 – 2 days	3 – 6 days	7 – 12 days	13 – 26 days	27 – 90 days	P value
From 2	1.00 (0.71 – 1.41)	1.45 (1.08 – 1.97)	1.98 (1.47 – 2.68)	2.06 (1.49 – 2.85)	1.77 (1.21 – 2.60)	0.01
From 3	1.02 (0.62 – 1.69)	1.21 (0.77 – 1.90)	1.48 (0.94 – 2.35)	1.21 (0.74 – 2.00)	1.09 (0.61 – 1.97)	0.61
From 4	0.74 (0.45 – 1.24)	0.70 (0.45 – 1.1)	0.71 (0.46 – 1.12)	1.24 (0.8 – 1.96)	1.13 (0.66 – 1.96)	0.20
From 5	0.94 (0.62 – 1.43)	0.95 (0.65 – 1.4)	0.81 (0.55 – 1.21)	0.85 (0.56 – 1.30)	1.06 (0.66 – 1.72)	0.87
From 6	1.29 (0.81 – 2.06)	0.82 (0.52 – 1.29)	0.76 (0.48 – 1.22)	0.88 (0.55 – 1.44)	0.67 (0.38 – 1.20)	0.27
Hospital type (Type 1, ref)						
Type 2	1.11 (0.79 – 1.56)	1.12 (0.83 – 1.52)	1.23 (0.91 – 1.68)	0.97 (0.71 – 1.36)	0.95 (0.65 – 1.40)	0.71
Type 3	0.59 (0.17 – 2.05)	0.92 (0.34 – 2.52)	1.32 (0.48 – 3.67)	0.95 (0.31 – 2.91)	0.91 (0.23 – 3.56)	0.92
Type 4	0.97 (0.39 – 2.39)	0.88 (0.41 – 1.90)	1.13 (0.55 – 2.34)	1.27 (0.61 – 2.64)	0.97 (0.41 – 2.29)	0.97
Region (Region 0, ref)						
Region 1	0.98 (0.61 – 1.58)	0.82 (0.53 – 1.27)	1.42 (0.91 – 2.23)	1.45 (0.89 – 2.38)	1.42 (0.81 – 2.52)	0.20
Region 2	1.02 (0.45 – 2.33)	0.77 (0.38 – 1.57)	0.92 (0.46 – 1.83)	1.14 (0.56 – 2.35)	0.69 (0.29 – 1.65)	0.77
Region 3	1.21 (0.76 – 1.96)	0.86 (0.56 – 1.35)	0.90 (0.57 – 1.45)	1.14 (0.69 – 1.88)	1.53 (0.87 – 2.70)	0.37
Region 4	0.83 (0.34 – 2.06)	0.73 (0.33 – 1.61)	0.77 (0.36 – 1.70)	0.79 (0.35 – 1.77)	0.91 (0.37 – 2.28)	0.95
Region 5	0.75 (0.2 – 2.89)	0.94 (0.32 – 2.82)	2.45 (0.88 – 6.84)	1.71 (0.54 – 5.41)	1.59 (0.39 – 6.49)	0.46
Region 6	0.24 (0.03 – 2.26)	0.68 (0.14 – 3.41)	1.36 (0.30 – 6.32)	1.69 (0.30 – 9.53)	0.84 (0.08 – 8.72)	0.75
Region 7	1.27 (0.64 – 2.55)	0.98 (0.51 – 1.90)	0.81 (0.40 – 1.66)	1.45 (0.69 – 3.08)	0.77 (0.3 – 2.00)	0.49

© 2019 Murugan R et al. *JAMA Network Open.*

eTable 5. Sensitivity Analyses of NUF Rate and Mortality

Characteristic	Net Ultrafiltration Rate (mL/kg/hr)	No. of patients	Adjusted Hazard Ratios[s]	95%C[t]	Time Interval (days)[u]	P value[v]
Alternative lower NUF threshold[a]	>1.75 vs. <1.01	1,341	1.62	1.12 – 2.34	27 – 90	0.01
	1.01–1.75 vs. <1.01		1.11	0.81 – 1.53	13 – 26	0.54
Alternative higher NUF threshold[b]	>1.75 vs. <1.01	1,341	1.68	1.16 – 2.43	27 – 90	0.01
	1.01–1.75 vs. <1.01		1.27	0.94 – 1.73	13 – 26	0.25
Using NUF for the first 72 hours of CVVHDF only[c]	>1.65 vs. <0.82	1,338	1.72	1.20 – 2.47	27 – 90	0.01
	0.82–1.65 vs. <0.82		1.32	0.97 – 1.79	13 – 26	0.25
Using maximum NUF values[d]	>2.66 vs. <1.57	1,341	1.95	1.44 – 2.65	7 – 12	<0.001
	1.57 – 2.66 vs. <1.57	1,341	1.25	0.90 – 1.72	13 – 26	0.12
Excluding 92 patients with no fluid removal[e]	>1.75 vs. <1.01	1,255	1.57	1.08 – 2.28	27 – 90	0.02
	1.01–1.75 vs. <1.01		1.12	0.83 – 1.52	3 – 6	0.42
Including patients with missing treatment hours by assigning them NUF of 0 mL/kg/hr[f]	>1.75 vs. <1.01	1,366	1.60	1.12 – 2.29	27 – 90	0.02
	1.01–1.75 vs. <1.01		1.12	0.82 – 1.53	13 – 26	0.58
Including patients with missing treatment hours by assigning them NUF of 1.43 mL/kg/hr[g]	>1.75 vs. <1.01	1,366	1.65	1.15 – 2.38	27 – 90	0.01
	1.01–1.75 vs. <1.01		1.19	0.87 – 1.63	13 – 26	0.43
Increasing time interval in Gray's model by 1 day[h]	>1.75 vs. <1.01	1,341	1.68	1.16 – 2.44	28 – 90	0.006
	1.01–1.75 vs. <1.01		1.17	0.85 – 1.63	14 – 27	0.37
Decreasing time interval in Gray's model by 1 day[i]	>1.75 vs. <1.01	1,341	1.63	1.14 – 2.33	26 – 90	0.006
	1.01–1.75 vs. <1.01		1.17	0.89 – 1.55	2 – 5	0.58
Using baseline individual organ SOFA scores[j]	>1.75 vs. <1.01	1,341	1.64	1.14 – 2.38	27 – 90	0.01
	1.01–1.75 vs. <1.01		1.19	0.86 – 1.63	13 – 26	0.56

© 2019 Murugan R et al. *JAMA Network Open.*

Characteristic	Net Ultrafiltration Rate (mL/kg/hr)	No. of patients	Adjusted Hazard Ratio[s]	95%C[t]	Time Interval (days)[u]	P value[v]
Adjusting for red cell, fresh frozen plasma, platelets, cryoprecipitate, 20% albumin use and cumulative protein supplementation[k]	>1.75 vs. <1.01	1,341	1.60	1.14 – 2.25	0 – 2	0.003
	1.01–1.75 vs. <1.01		1.20	0.90 – 1.60	3 – 6	0.28
Using cumulative fluid balance including NUF volume in the output fluid calculation[l]	>1.75 vs. <1.01	1,341	1.74	1.21 – 2.51	27 – 90	0.004
	1.01–1.75 vs. <1.01		1.19	0.87 – 1.63	13 – 26	0.60
Excluding cumulative fluid balance from the model [m]	>1.75 vs. <1.01	1,341	1.72	1.20 – 2.47	27 – 90	0.005
	1.01–1.75 vs. <1.01	1,341	1.18	0.86 – 1.62	13 – 26	0.62
Patients receiving CVVHDF for <3 days[n]	>1.75 vs. <1.01	480	1.42	0.93 – 2.19	0 – 2	0.28
	1.01–1.75 vs. <1.01		1.41	0.94 – 2.11	0 – 2	0.35
Patients receiving CVVHDF for ≥3 days[0]	>1.75 vs. <1.01	861	1.99	1.22 – 3.23	33 – 90	0.029
	1.01–1.75 vs. <1.01		1.06	0.69 – 1.61	17 – 33	0.46
Patients receiving CVVHDF for <5 days[p]	>1.75 vs. <1.01	803	1.40	0.92 – 2.13	0 – 2	0.44
	1.01–1.75 vs. <1.01		1.51	1.07 – 2.13	2 – 4	0.05
Patients receiving CVVHDF for ≥5 days[q]	>1.75 vs. <1.01	538	1.93	1.00 – 3.75	31 – 90	0.28
	1.01–1.75 vs. <1.01		1.24	0.70 – 2.20	18 – 31	0.32
Patients with daily mean negative fluid balance[r]	>2.03 vs. <1.39	683	2.71	1.56 – 4.69	0 – 6	<0.001
	1.39 – 2.03 vs. <1.39		1.90	1.09 – 3.30	0 – 6	0.14

© 2019 Murugan R et al. *JAMA Network Open.*

[a] The threshold of NUF was lowered by 0.05mls/kg/hr defining the three groups as follows: <0.96 mL/kg/hr; 0.96 - 1.70 mL/kg/hr and >1.70 mL/kg/hr.

[b] The threshold of NUF was increased by 0.05 mL/kg/hr defining the three groups as follows: <1.06 mL/kg/hr; 1.06 -1.80 mL/kg/hr and >1.80 mL/kg/hr.

[c] NUF was calculated only for the first 72 hours of treatment with CVVHDF.

[d] Using maximum NUF rate for each patient the threshold values for the three groups were <1.56 mL/kg/hr, 1.56 – 2.66 mL/kg/hr and, >2.66 mL/kg/hr.

[e] Excluding 92 patients with fluid removal <0.01 mL/kg/hr from the cohort.

[f] Patients with missing data (n=31) on treatment hours were assigned an NUF rate of 0 mL/kg/hr.

[g] Patients with missing data (n=31) on treatment hours were assigned a mean NUF rate of 1.43 mL/kg/hr in the study population.

[h] The time intervals from and the nodes in the Gray's model was increased by 1 day defining new time intervals as follows: 0-3, 4-7, 8-13, 14-27 and 28-90 days.

[i] The time intervals from and the nodes in the Gray's model was decreased by 1 day defining new time intervals as follows: 0-1,2-5, 6-11, 12-25 and 26-90 days.

[j] Model adjusted using individual baseline liver, coagulation, and respiratory SOFA score instead of total SOFA score.

[k] Model adjusted for additional covariates including use of red cells, fresh frozen plasma, platelets, cryoprecipitate, 20% albumin, and cumulative protein supplementation during CVVHDF.

[l] Model adjusted for cumulative fluid balance calculated without excluding the NUF volume from the output fluid calculation.

© 2019 Murugan R et al. *JAMA Network Open.*

[m] After excluding cumulative fluid balance from the model.

[n] Patients who were treated with CVVHDF for less than 3 days.

[o] Patients who were treated with CVVHDF for 3 or more days.

[p] Patients who were treated with CVVHDF for less than 5 days.

[q] Patients who were treated with CVVHDF for 5 or more days.

[r] Patients who had negative mean daily fluid balance throughout ICU stay.

[s] Shown are the highest adjusted hazard ratio from the Gray's model.

[t] Shown are 95% CI corresponding to the highest adjusted hazard ratio from the Gray's model.

[U] Shown are the time interval corresponding to the highest adjusted hazard ratio from the Gray's model.

[V] Shown are the overall P values for all time intervals from the Gray's model.

© 2019 Murugan R et al. *JAMA Network Open.*

eTable 6. Association of NUF Rate With Risk-Adjusted Mortality From Logistic Regression

All Covariates	Unadjusted Odds Ratio (95%CI)	P Value	Adjusted Odds Ratio (95%CI)	P value
<1.01 mL/kg/hr (ref)				
>1.75 mL/kg/hr	1.21 (0.93 – 1.58)	0.03	1.25 (0.91 - 1.73)	0.02
1.01 – 1.75 mL/kg/hr	0.84 (0.65 – 1.11)		0.81 (0.59 – 1.11)	
Age , years, <53.25 (ref)				
53.25 – <63.58	1.81 (1.27 – 2.57)	<0.001	1.57 (1.04 – 2.39)	0.003
63.58 – <71.09	1.75 (1.23 – 2.57)		1.67 (1.09 – 2.56)	
71.09 – <77.72	1.94 (1.37 – 2.77)		2.10 (1.35 – 3.26)	
≥77.72	2.15 (1.54 – 3.05)		2.37 (1.51 – 3.71)	
eGFR	1.00 (1.00 – 1.00)	0.91	1.00 (1.00 – 1.01)	0.48
Female sex	1.07 (0.86 – 1.35)	0.53	1.11 (0.85 – 1.44)	0.45
Mechanical Ventilation	2.15 (1.66 – 2.78)	<0.001	1.48 (1.04 – 2.11)	0.03
APACHE III score, <82 (ref)				
82 - <95	1.52 (1.06 – 2.19)	<0.001	1.35 (0.89 – 2.05)	<0.001
95 - <107	1.53 (1.06 – 2.22)		1.41 (0.91 – 2.17)	
107 - <122	2.20 (1.54 – 3.16)		1.79 (1.15 – 2.77)	
≥122	3.61 (2.51 – 5.12)		2.83 (1.79 – 4.47)	
Organ edema	1.12 (0.90 – 1.40)	0.29	0.95 (0.73 – 1.22)	0.70
Sepsis	1.33 (1.07 – 1.65)	0.01	0.83 (0.63 – 1.09)	0.20
Time from ICU admission to study enrollment	1.00 (1.00 – 1.00)	<0.001	1.00 (1.00 – 1.00)	<0.001
Total SOFA score at randomization	1.16 (1.12 – 2.21)	<0.001	1.01 (0.95 – 1.07)	0.72
Mean Daily Cardiovascular SOFA score	1.85 (1.69 – 2.05)	<0.001	1.77 (1.57 – 1.99)	<0.001
Mean fluid balance	1.00 (1.00 – 1.00)	0.14	1.00 (1.00 – 1.00)	0.46
Duration of study treatment	1.00 (0.99 – 1.02)	0.61	1.00 (0.98 – 1.02)	0.63
Source of admission (From 6, ref)				
From 1	1.03 (0.70-1.52)	<0.001	1.34 (0.83 – 2.17)	<0.001
From 2	1.96 (1.35-2.86)		2.97 (1.87 – 4.71)	
From 3	1.25 (0.77-2.06)		1.68 (0.94 – 2.99)	
From 4	0.92 (0.58-1.47)		1.21 (0.70 – 2.09)	
From 5	1.14 (0.74-1.74)		1.13 (0.69 – 1.83)	
Hospital type 0 (ref)				
Hospital type 1	1.55 (0.82-2.92)	0.38	1.09 (0.51 – 2.34)	0.97
Hospital type 2	1.58 (0.82-3.05)		1.09 (0.50 – 2.37)	

© 2019 Murugan R et al. *JAMA Network Open.*

All Covariates	Unadjusted Odds Ratio (95%CI)	P Value	Adjusted Odds Ratio (95%CI)	P value
Hospital type 3	0.93 (0.31-2.78)		0.87 (0.25 – 3.00)	
Region 7 (ref)				
Region 0	0.72 (0.39-1.52)	0.04	1.08 (0.5 – 2.32)	0.15
Region 1	0.96 (0.55-1.71)		1.53 (0.80 – 2.93)	
Region 2	0.47 (0.23-0.97)		0.74 (0.33 – 1.70)	
Region 3	0.88 (0.50-1.56)		1.30 (0.67 – 2.54)	
Region 4	0.43 (0.20-0.94)		0.75 (0.31 – 1.83)	
Region 5	0.87 (0.28-2.69)		1.77 (0.47 – 6.70)	
Region 6	1.12 (0.21-6.07)		1.22 (0.18 – 8.11)	

© 2019 Murugan R et al. *JAMA Network Open.*

eTable 7. Association of NUF Rate With Risk-Adjusted Mortality Using NUF as a Continuous Variable From Logistic Regression

All Covariates	Unadjusted Odds Ratio (95%CI)	P Value	Adjusted Odds Ratio (95%CI)	P value
NUF rate per 0.50 mL/kg/hr increase	1.098 (0.90 – 1.23)	0.09	1.07 (1.00 – 1.15)	0.043
Age , years, <53.25 (ref)				
53.25 – <63.58	1.81 (1.27-2.57)	<0.001	1.63 (1.07 – 2.48)	0.003
63.58 – <71.09	1.75 (1.23-2.49)		1.69 (1.10 – 2.61)	
71.09 – <77.72	1.94 (1.37-2.77)		2.10 (1.35 – 3.26)	
≥77.72	2.15 (1.54-3.05)		2.40 (1.53 – 3.76)	
eGFR	1.00 (1.00-1.00)	0.91	1.00 (0.99 – 1.00)	0.49
Female sex	1.07 (0.86-1.35)	0.53	1.09 (0.84 – 1.43)	0.48
Mechanical Ventilation	2.15 (1.66-2.78)	<0.001	1.47 (1.04 – 2.09)	0.02
APACHE III score, <82 (ref)				
82 - <95	1.52 (1.06-2.19)	<0.001	1.34 (0.88 – 2.04)	<0.001
95 - <107	1.53 (1.06-2.22)		1.41 (0.91 – 2.17)	
107 - <122	2.20 (1.54-3.16)		1.78 (1.15 – 2.76)	
≥122	3.61 (2.51-5.12)		2.80 (1.77 – 4.42)	
Organ edema	1.12 (0.90-1.40)	0.29	0.94 (0.73 – 1.22)	0.65
Sepsis	1.33 (1.07-1.65)	0.01	0.83 (0.63– 1.08)	0.17
Time from ICU admission to study enrollment	1.00 (1.00-1.00)	<0.001	1.00 (1.00 – 1.00)	<0.001
Total SOFA score at enrollment	1.16 (1.12-2.21)	<0.001	1.01 (0.95 – 1.07)	0.74
Mean daily cardiovascular SOFA score	1.85 (1.69-2.05)	<0.001	1.79 (1.58 – 2.02)	<0.001
Mean fluid balance	1.00 (1.00-1.00)	0.14	1.00 (1.00 – 1.00)	0.49
Duration of study treatment	1.00 (0.99-1.02)	0.61	1.00 (0.98 – 1.02)	0.70
Source of admission (ref)				
From 1	1.03 (0.70-1.52)	<0.001	1.37 (0.85 – 2.20)	<0.001
From 2	1.96 (1.35-2.86)		3.03 (1.91 – 4.80)	
From 3	1.25 (0.77-2.06)		1.63 (0.91 – 2.90)	
From 4	0.92 (0.58-1.47)		1.24 (0.72 – 2.15)	
From 5	1.14 (0.74-1.74)		1.16 (0.72 – 1.88)	
Hospital type 0 (ref)				
Hospital type 1	1.55 (0.82-2.92)	0.38	1.06 (0.50 – 2.25)	0.97
Hospital type 2	1.58 (0.82-3.05)		1.05 (0.48 – 2.27)	
Hospital type 3	0.93 (0.31-2.78)		0.84 (0.25 – 2.88)	
Region 7 (ref)				
Region 0	0.72 (0.39-1.52)	0.04	1.05 (0.49 – 2.26)	0.20
Region 1	0.96 (0.55-1.71)		1.45 (0.76 – 2.77)	

© 2019 Murugan R et al. *JAMA Network Open.*

All Covariates	Unadjusted Odds Ratio (95%CI)	P Value	Adjusted Odds Ratio (95%CI)	P value
Region 2	0.47 (0.23-0.97)		0.74 (0.33 – 1.70)	
Region 3	0.88 (0.50-1.56)		1.23 (0.63 – 2.39)	
Region 4	0.43 (0.20-0.94)		0.71 (0.29 – 1.73)	
Region 5	0.87 (0.28-2.69)		1.69 (0.45 – 6.35)	
Region 6	1.12 (0.21-6.07)		1.29 (0.20 – 8.53)	

© 2019 Murugan R et al. *JAMA Network Open.*

eTable 8. Subgroup Analyses of NUF Rate and Mortality

Subgroup[a]	Net Ultrafiltration Rate (mL/kg/hr)	No. of patients	Adjusted Hazard Ratio[b]	95%CI[c]	Time Interval[d]	P Value[e]	Interaction P Value
Organ edema	>1.75 vs. <1.01	581	1.61	1.01 – 2.55	3 – 7	0.13	0.61
	1.01–1.75 vs. <1.01		1.18	0.74 – 1.89	3 – 7	0.50	
No organ edema	>1.75 vs. <1.01	760	1.75	1.06 – 2.88	28 – 90	0.05	
	1.01–1.75 vs. <1.01		1.24	0.84 – 1.83	7 – 14	0.63	
Sepsis	>1.75 vs. <1.01	652	2.19	1.26 – 3.80	26 – 90	0.05	0.51
	1.01–1.75 vs. <1.01		1.07	0.68 – 1.70	13 – 26	0.99	
No sepsis	>1.75 vs. <1.01	689	1.72	1.14 – 2.59	7 – 14	0.06	
	1.01–1.75 vs. <1.01		1.36	0.87 – 2.13	14 – 29	0.46	
eGFR <60 mL/min/1.73m^2	>1.75 vs. <1.01	759	1.37	0.85 – 2.20	27 – 90	0.29	0.55
	1.01–1.75 vs. <1.01		1.24	0.81 – 1.91	0 – 3	0.36	
eGFR ≥60mL/min/1.73 m^2	>1.75 vs. <1.01	582	2.30	1.21 – 4.38	29 – 90	0.03	
	1.01–1.75 vs. <1.01		1.63	0.94 – 2.81	14 – 29	0.43	
Cardiovascular SOFA score <3	>1.75 vs. <1.01	371	1.31	0.67 – 2.56	17 – 42	0.57	0.22
	1.01–1.75 vs. <1.01		1.56	0.78 – 3.11	0 – 5	0.52	
Cardiovascular SOFA score ≥ 3	>1.75 vs. <1.01	970	1.89	1.27 – 2.81	22 – 90	0.01	
	1.01–1.75 vs. <1.01		1.06	0.77 – 1.45	2 – 5	0.94	
High intensity RRT	>1.75 vs. <1.01	657	2.22	1.35 – 3.66	25 – 90	0.002	0.50
	1.01–1.75 vs. <1.01		1.45	0.95 – 2.20	3 – 6	0.25	
Low intensity RRT	>1.75 vs. <1.01	684	1.50	0.94 – 2.40	14 – 31	0.38	
	1.01–1.75 vs. <1.01		1.46	0.94 – 2.67	14 – 31	0.45	

[a] Prespecified subgroup of patients defined at study enrollment in the RENAL trial.

[b] Shown are the highest adjusted hazard ratio from the Gray's model.

© 2019 Murugan R et al. *JAMA Network Open.*

[c] Shown are 95% CI corresponding to the highest adjusted hazard ratio from the Gray's model.

[d] Shown are the time interval from the Gray's model corresponding to the highest adjusted hazard ratio.

[e] Shown are the overall P value for all time intervals from the Gray's model.

© 2019 Murugan R et al. *JAMA Network Open.*

eFigure 1. Distributions of Imputed and Unimputed Premorbid Serum Creatinine and Estimated Glomerular Filtration Rate

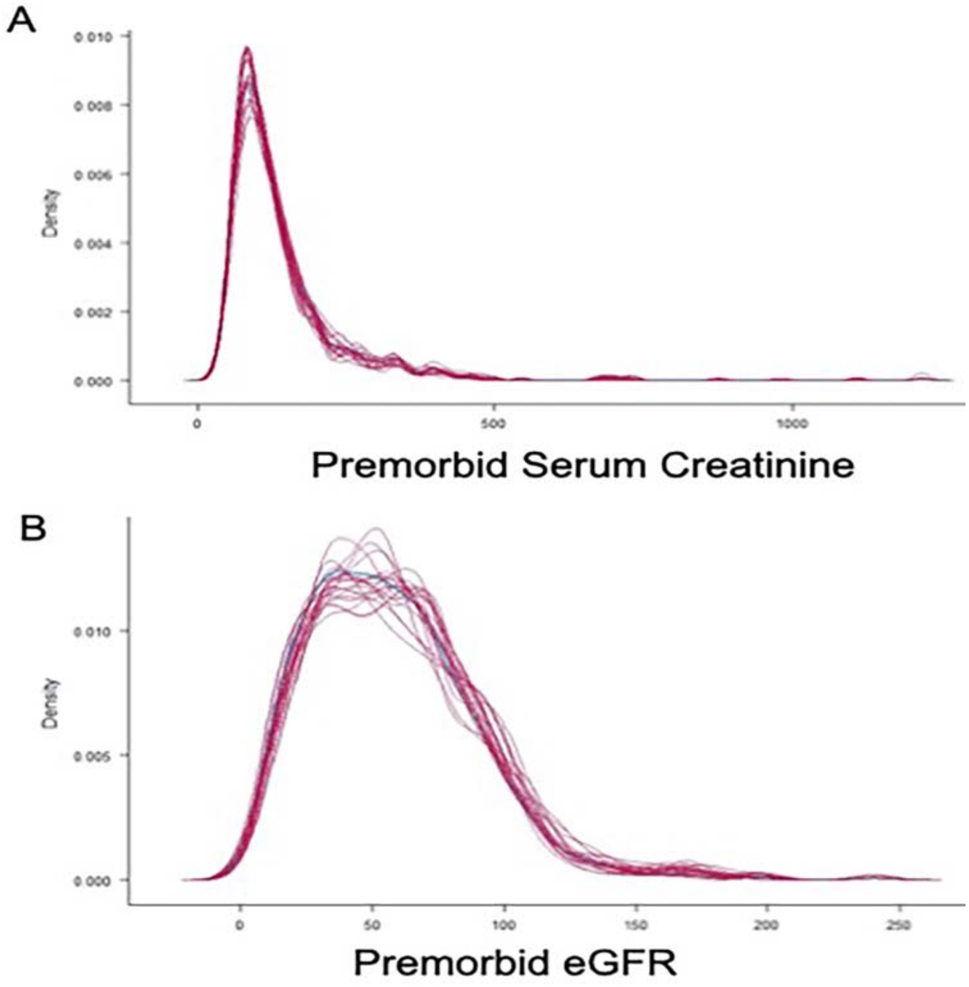

Figure showing distribution of imputed (red) and unimputed (blue) premorbid serum creatinine (A) and the corresponding premorbid estimated glomerular filtration rate (eGFR) (B). There was no difference in distribution of imputed and unimputed premorbid serum creatinine values (P=0.29) and the corresponding estimated premorbid glomerular filtration rate (P=0.13).

© 2019 Murugan R et al. *JAMA Network Open.*

eFigure 2. Association of NUF Rate With Crude 90-Day Mortality

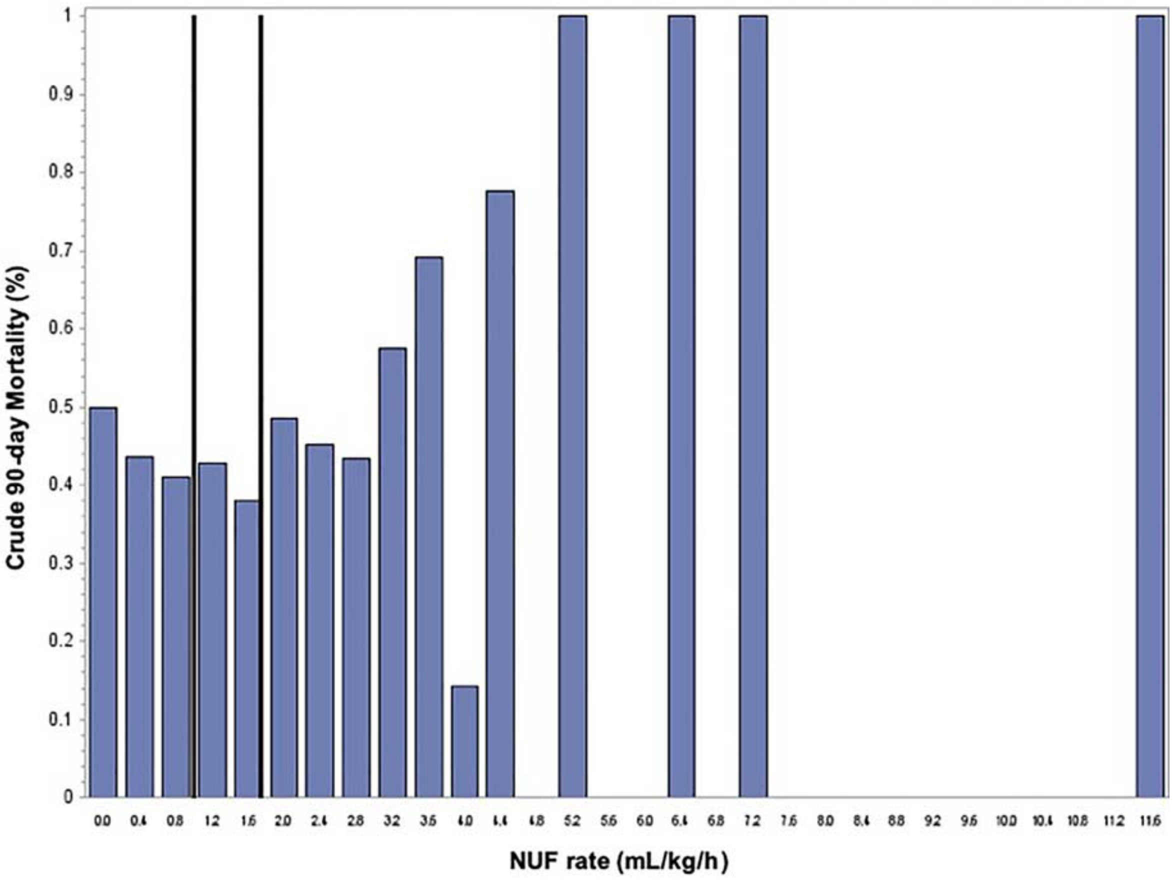

X -axis represents the NUF rate in mL/kg/hr. Y-axis represents the corresponding crude 90-day mortality in percentage. The black bar on the left represents the 25th percentile on the distribution corresponding to the NUF of 1.01 mL/kg/hr and the bar on the right represents the 75th percentile on the distribution corresponding to the NUF rate of of 1.75 mL/kg/hr.

© 2019 Murugan R et al. *JAMA Network Open.*

eFigure 3. Violation of Proportional Hazards Assumptions by NUF Rate Variable

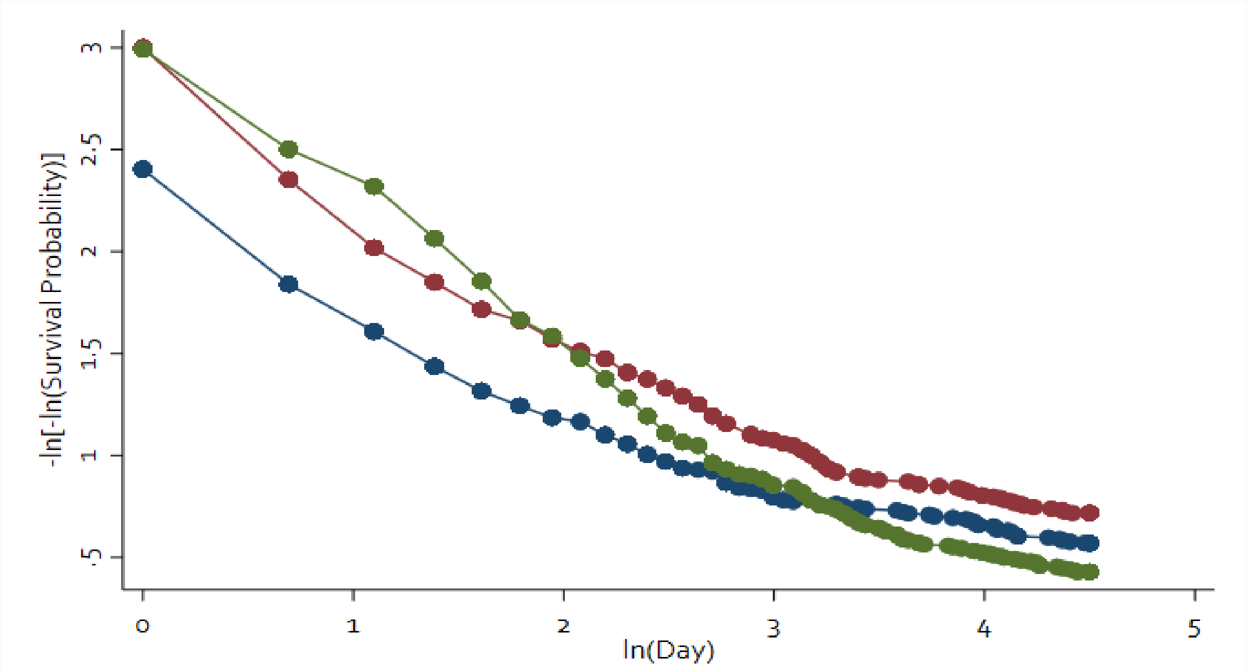

Log-log plots showing association between survival probability and duration of time in natural logarithm function for NUF rate variable <1.01 mL/kg/hr (blue), 1.01 - 1.75 mL/kg/hr (red) and >1.75 mL/kg/hr (green). The NUF rate variable >1.75 mL/kg/hr (green) violates proportionality assumptions and thus could not be fitted using the traditional Cox model, as the model assumes that the proportional hazards stay constant over time.

© 2019 Murugan R et al. *JAMA Network Open.*

eFigure 4. Distribution of Propensity Scores for Patients Who Received NUF Rate >1.75 mL/kg/hr Among Matched (N=405) and Unmatched Patients (N=532)

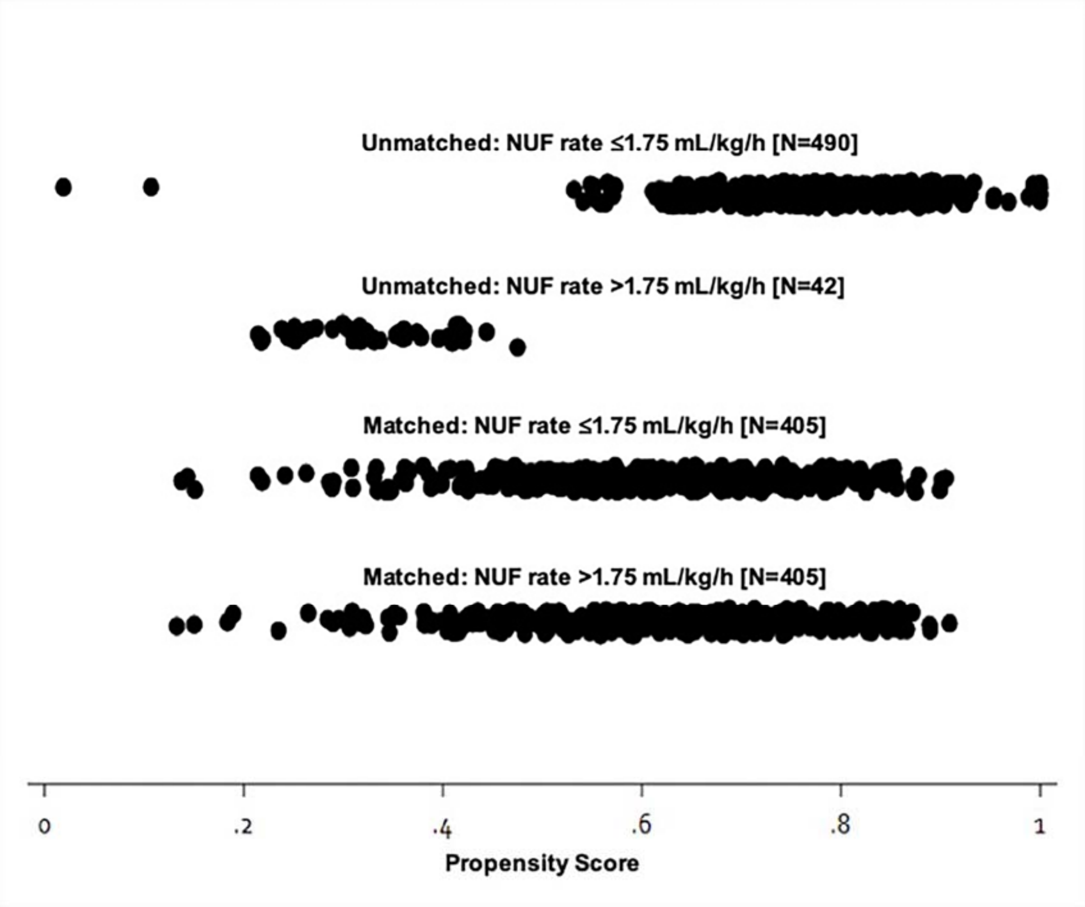

© 2019 Murugan R et al. *JAMA Network Open.*

eFigure 5. Logistic Regression Model Calibration and Discrimination

A: Model Calibration

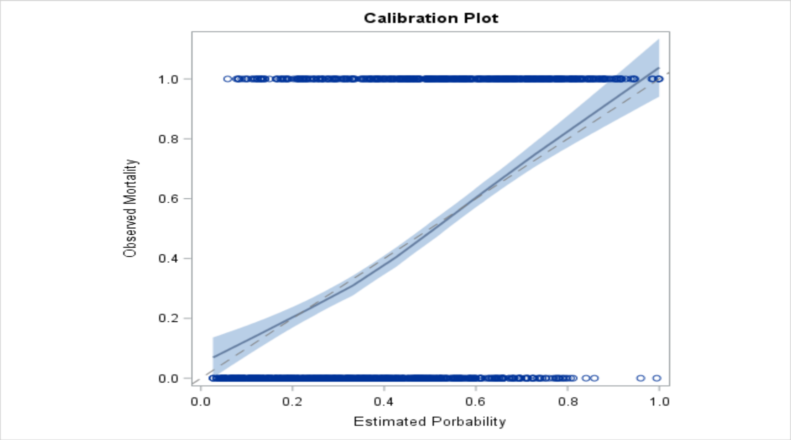

The calibration plot above shows observed versus predicted probability of death (blue line). The calibration belt shows the 95% confidence intervals (shaded light blue area) along the observed rates, which the upper and lower bounds do not cross the blue line over the range of expected probabilities. The belt and the Hosmer-Lemeshow statistic (P=0.18) confirm adequate calibration for the risk-adjustment model.

B: Model Discrimination

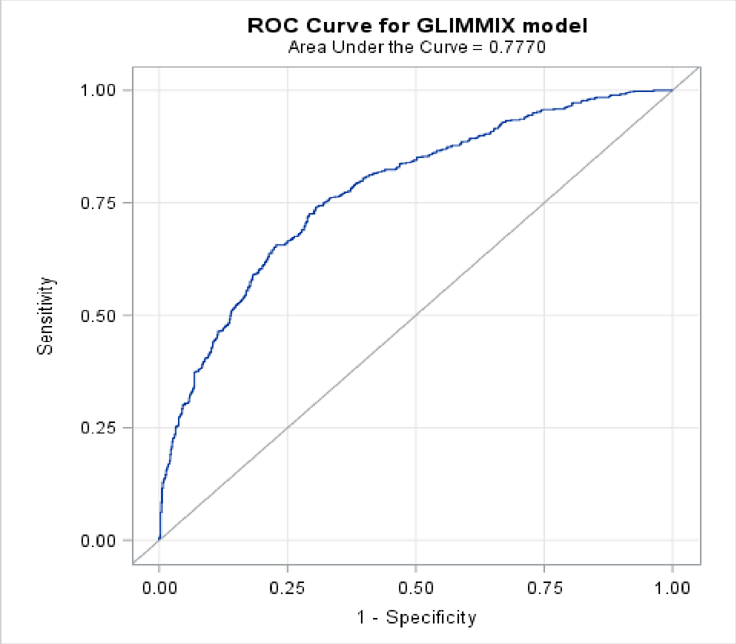

© 2019 Murugan R et al. *JAMA Network Open.*

eFigure 6. Crude 90-Day Mortality and Predicted Risk of Death by NUF Rate

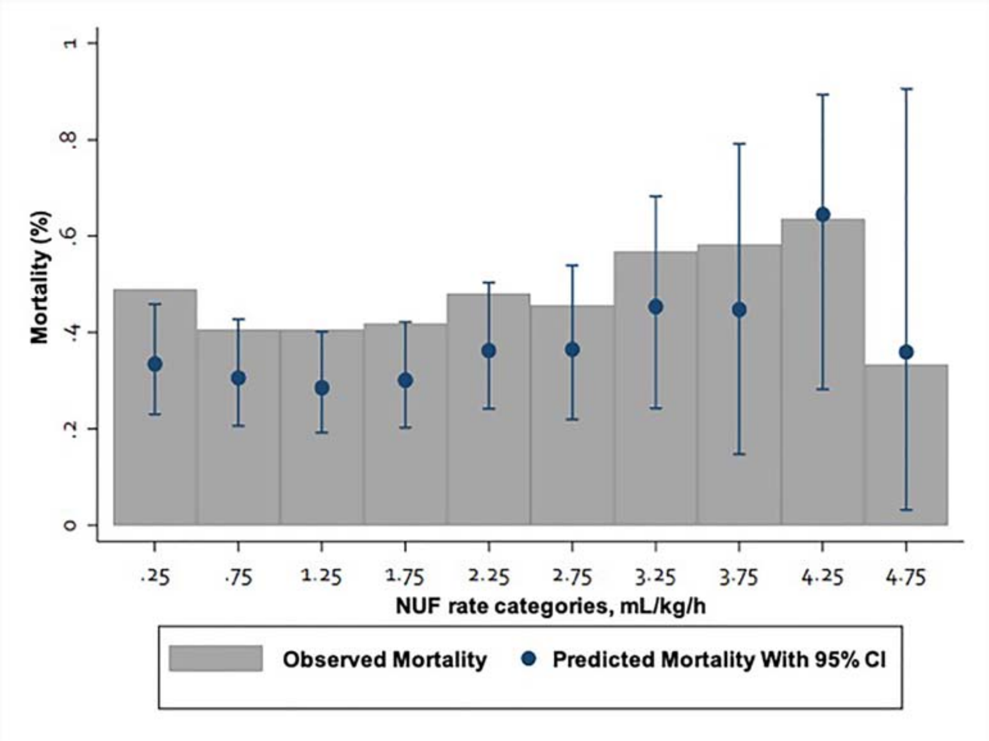

Crude 90 day mortality (bars) and the predicted risks of death with 95%CIs (blue line with error bars). Predicted risks derive from model adjusted for age category, female sex, APACHE-III score category 24 hours prior to study enrollment, presence of organ edema, presence of sepsis, use of mechanical ventilation, time from ICU admission to study enrollment, premorbid eGFR, source of admission to ICU either from the emergency room, operating room, or transfer from other ICU, clustering by region and hospital type; mean daily cardiovascular SOFA score after initiation of CVVHDF; cumulative daily fluid balance during ICU stay and duration of renal replacement therapy in days.

As an interpretive example, for a typical patient with average age and level of acuity enrolled in the RENAL trial, NUF rate of 1 mL/kg/hr will have 30.6% predicted risk of death. In contrast, a patient receiving NUF rate of 4.5 mL/kg/hr will have a 64.5% predicted risk of death.

© 2019 Murugan R et al. *JAMA Network Open.*

eFigure 7. Association of NUF Rate With 90-Day Survival

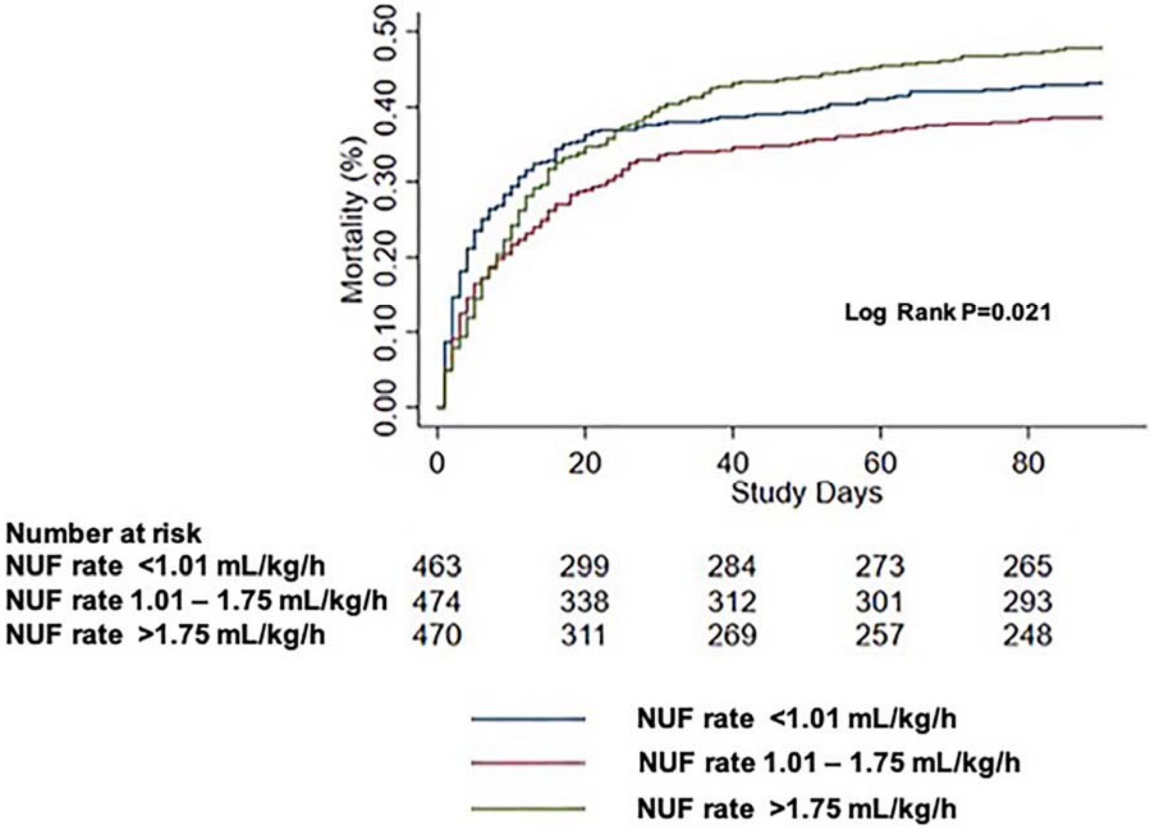

The Kaplan Meier failure plots by NUF rate for probability of death over 90 days from study enrollment in the overall cohort (n=1,434). Blue line represents NUF rate <1.01 mL/kg/hr, red line represents rate of 1.01 - 1.75 mL/kg/hr and green line represents rate >1.75 mL/kg/hr. The probability of death was highest in the NUF rate >1.75 mL/kg/hr compared with <1.01 mL/kg/hr and 1.01 – 1.75 mL/kg/hr NUF groups (Log Rank P=0.02).

© 2019 Murugan R et al. *JAMA Network Open.*

eReferences

1. Bellomo R, Cass A, Cole L, et al; RENAL Replacement Therapy Study Investigators. Intensity of continuous renal-replacement therapy in critically ill patients. *N Engl J Med.* 2009;361(17):1627-1638.
2. Bellomo R, Cass A, Cole L, et al; RENAL Replacement Therapy Study Investigators. An observational of study fluid balance and patient outcomes in the Randomized Evaluation of Normal vs. Augmented Level of Replacement Therapy trial. *Crit Care Med.* 2012;40(6):1753-1760.
3. Little RJA, Rubin D. *Statistical Analysis with Missing Data.* Hoboken, NJ: John Wiley and Sons; 2002.
4. Donders AR, van der Heijden GJ, Stijnen T, Moons KG. Review: a gentle introduction to imputation of missing values. *J Clin Epidemiol.* 2006;59(10):1087-1091.
5. Siew ED, Peterson JF, Eden SK, Moons KG, Ikizler TA, Matheny ME. Use of multiple imputation method to improve estimation of missing baseline serum creatinine in acute kidney injury research. *Clin J Am Soc Nephrol.* 2013;8(1):10-18.
6. Rubin D. Multiple impuation after 18+ years. *J Am Stat Assoc.* 1996;91:473-489.
7. Buuren SG-O, K. mice: Multivariate Imputation by Chained Equations in R. *Journal of Statistical Software.* 2011;45(3):1-67.
8. Zavada J, Hoste E, Cartin-Ceba R, et al. A comparison of three methods to estimate baseline creatinine for RIFLE classification. *Nephrol Dial Transplant.* 2010;25(12):3911-3918.
9. Balakumar V, Murugan R, Sileanu FE, Palevsky P, Clermont G, Kellum JA. Both Positive and Negative Fluid Balance May Be Associated With Reduced Long-Term Survival in the Critically Ill. *Crit Care Med.* 2017;45(8):e749-e757.
10. Murugan R, Balakumar V, Kerti SJ, et al. Net ultrafiltration intensity and mortality in critically ill patients with fluid overload. Crit Care. 2018;22(1):223.
11. Cox DR. Regression Models and Life-Tables. *J R Stat Soc B.* 1972;34(2):187.
12. Murugan R, Karajala-Subramanyam V, Lee M, et al. Acute kidney injury in non-severe pneumonia is associated with an increased immune response and lower survival. *Kidney Int.* 2010;77(6):527-535.
13. Gray RJ. Flexible Methods for Analyzing Survival Data Using Splines, with Applications to Breast Cancer Prognosis. *Journal of the American Statistical Association.* 1992;87(420):942-951.
14. Gray RJ. Spline-based tests in survival analysis. *Biometrics.* 1994;50(3):640-652.
15. Kasal J, Jovanovic Z, Clermont G, et al. Comparison of Cox and Gray's survival models in severe sepsis. *Crit Care Med.* 2004;32(3):700-707.
16. Valenta Z, Weissfeld L. Estimation of the survival function for Gray's piecewise-constant time-varying coefficients model. *Stat Med.* 2002;21(5):717-727.
17. Rizopoulos D. Dynamic predictions and prospective accuracy in joint models for longitudinal and time-to-event data. *Biometrics.* 2011;67(3):819-829.
18. Wulfsohn MS, Tsiatis AA. A joint model for survival and longitudinal data measured with error. *Biometrics.* 1997;53(1):330-339.

© 2019 Murugan R et al. *JAMA Network Open.*
